# The added value of a family-centered approach to optimize infants’ social-emotional development: A quasi-experimental study

**DOI:** 10.1371/journal.pone.0187750

**Published:** 2017-12-21

**Authors:** Sijmen A. Reijneveld, Margriet Hielkema, Roy E. Stewart, Andrea F. de Winter

**Affiliations:** Department of Health Sciences, University Medical Center Groningen, University of Groningen, Groningen, the Netherlands; IRCCS E. Medea, ITALY

## Abstract

**Objective:**

Family-centered care (FCC) has been related to positive healthcare outcomes in pediatric care. Our aim was to assess whether an FCC approach also leads to better and earlier identification of social-emotional problems and less child psychosocial problems at age 18 months.

**Methods:**

In a quasi-experimental study within routine well-child care in the Netherlands, we compared those regions in which an FCC approach was implemented (FCC-JointStart) to those regions with “care-as-usual” (CAU), including all children. In all regions, professionals performed well-child visits (2–18 months) and assessed social-emotional problems, or risks developing these, by rating outcomes of assessments as “*not optimal*” or as “*a problem*.” We compared FCC-JointStart and CAU regarding the rates of newly identified (risks for) social-emotional problems, the pace of identification over time, and the child’s psychosocial wellbeing at eighteen months as measured by the Child Behavior Checklist (CBCL). For participants that received extra care, we compared FCC-JointStart and CAU regarding the severity of parent-reported problems. Parents were blinded, professionals were not.

**Results:**

5658 parents (68%) agreed to participate in the study. In the FCC-JointStart group, risks were identified more frequently, though differences were small (24.7% versus 22.0%, odds ratio (95%-confidence interval) adjusted for confounders: 1.44 (0.96; 2.18), Phi = .03). Risks were also identified earlier (p = .008), and additional care was provided to more severe cases than in CAU. Effect sizes *r* ranged from 0.17 (PSBC) to 0.22 (FAD). CBCL scores at 18 months did not differ between groups.

**Conclusions:**

FFC-JointStart may contribute to more and earlier identification of risks for social-emotional problems and of families that need additional care, but not to fewer child psychosocial problems at age 18 months.

**Trial registration:**

Netherlands Trial Register NTR2681

## Introduction

The importance of children’s social-emotional wellbeing for later life has been widely recognized.[1–3] As a consequence, multiple studies have focused on the identification of social-emotional problems in children[4–6], because children and their families may benefit from early intervention if social-emotional problems occur.[7–9] However, the early identification of social-emotional and psychosocial problems in children could be improved.[4,5,10]

Family-centered care (FCC) may help to optimize the early identification process. The key elements of FCC according to the American Academy of Pediatrics are described in Table 1.[11] FCC may optimize the early identification process by a number of characteristics. First it takes into account the expert view of parents about their child.[12,13] This may stimulate parents to express their view concerning the child’s development, and thus to disclose their concerns more easily, which can be beneficial for identification of potential problems.[14] Second, FCC may optimize early identification by taking into account the child in the context in which it grows up. This context highly determines the child’s development,[15] in addition to its genetic and biological make-up. Furthermore, FCC may also promote children’s social-emotional wellbeing through empowerment of the parents, which can enhance parents’ self-confidence and parenting skills.[16]

**Table 1:** Core principles of family-centered care according to the American Academy of Pediatrics.

1. Respecting each child and his or her family
2. Honoring racial, ethnic, cultural, and socioeconomic diversity and its effect on the family’s experience and perception of care
3. Recognizing and building on the strengths of each child and family, even in difficult and challenging situations and respecting different methods of coping
4. Supporting and facilitating choice for the child and family about approaches to care and support
5. Ensuring flexibility in organizational policies, procedures, and provider practices so services can be tailored to the needs, beliefs, and cultural values of each child and family
6. Sharing honest and unbiased information with families on an ongoing basis and in ways they find useful and affirming
7. Providing and/or ensuring formal and informal support (eg, family-to-family support) for the child and parent(s) and/or guardian(s) during pregnancy, childbirth, infancy, childhood, adolescence, and young adulthood
8. Collaborating with families at all levels of health care, in the care of the individual child and in professional education, policy making, and program development
9. Empowering each child and family to discover their own strengths, build confidence, and make choices and decisions about their health

FCC has been recognized as pivotal for the quality of preventive pediatric care, as reflected in guidelines like *Bright Futures* of the American Academy of Pediatrics.[17] In the Netherlands, an FCC approach (FCC-JointStart) has been implemented in Preventive Child Healthcare (PCH) for use in routine well-child visits. PCH is similar to well-child care in the US, but is free of charge for all families and is far reaching (>90%). FCC-JointStart consists of a family-centered way of communicating with parents (as further detailed in the Methods section), in combination with a checklist regarding the child’s social-emotional wellbeing and developmental context. We previously showed that FCC-JointStart leads to better attunement of care to parents’ preferences[18], and to the identification of those families who need additional care, as reflected by higher problem scores on a range of contextual problems.[19] However, there is lack of evidence on whether this type of FCC indeed leads to *earlier* identification and to better child outcomes, which are the core of well-child care.

Therefore, in this study our aim was first to assess whether FCC-JointStart leads to a better and earlier identification of social-emotional problems as compared to care-as-usual (CAU). Better and earlier identification regarded more identification, earlier identification, and identification of the more severe problems. Second, we assessed whether FCC-JointStart leads to fewer child psychosocial problems at age 18 months.

## Methods

### Design and setting

We conducted a non-blinded quasi-experimental study within a Dutch PCH organization, which implemented an FFC approach in one region, but not in others. This led to an intervention region (FCC-JointStart; 7 Well-Child Centers, WCC) and a care-as-usual (CAU; 5 WCC) region; WCC were assigned top-down to either condition. The FCC-JointStart and CAU regions were comparable regarding socio-demographic variables, including income, employment, ethnicity, and household composition. PCH professionals did also not differ between regions regarding gender, age, and years of experience. Randomization per child was not possible because professionals worked only in one of both regions. The Medical Ethics Committee of the University Medical Center Groningen approved our study and all participants provided written informed consent (METc2008.272 dated August 5, 2009). The authors confirm that they neither perform nor have performed any other trial for this intervention. Registration of the study in the trial register was delayed until the study had started because originally we did not think registration was needed as it involved a non-commercial intervention. Further details of the study design are described elsewhere.[20]

### Participants

**Fig 1:**
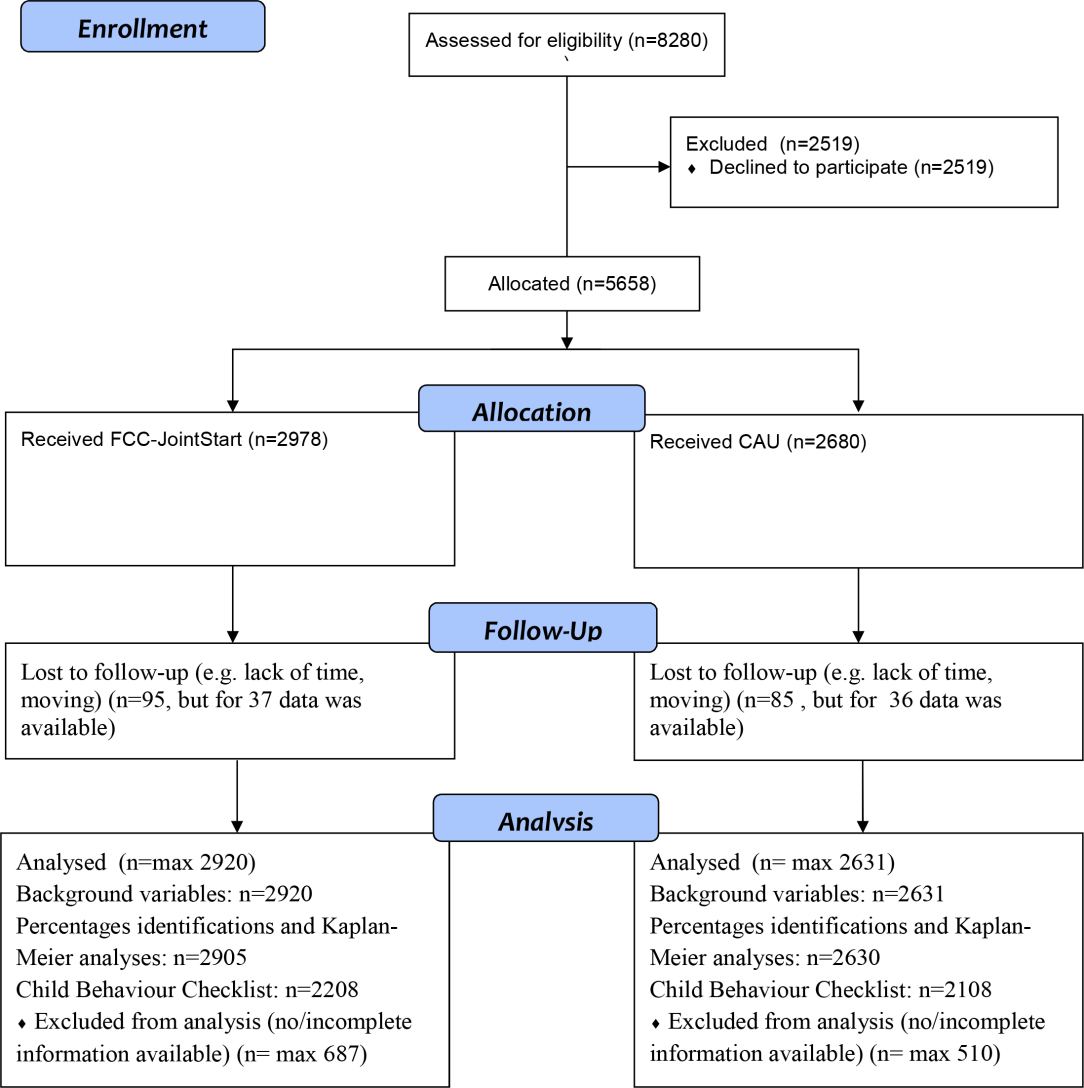
Flow of participants through the study.

Parents were eligible if they had sufficient mastery of the Dutch language and had a well-child PCH visit with their newborn child in the regions concerned (parts of the Dutch provinces of Drenthe and Overijssel). Between October 2009 and June 2011, PCH professionals, i.e. nurses and medical doctors, asked 8280 parents to participate, (84%) of all eligible parents, with no exclusions. Inclusion was ended because of obtaining the required number of case. Of those asked, 5658 (68%, in both regions) agreed to participate. Participants were followed for 18 months, with final follow-up assessments in December, 2012. Differences in background characteristics (employment status, educational level, country of birth and age of the parents) were small between parents who were and were not invited to participate, and between parents who agreed and refused to participate (Cramer’s V varying from .05 to .13). At 18 months, 5478 families (97%) were still participating; details are provided in Fig 1.

### Intervention group

FCC-JointStart was used during all routine well-child visits (from 2 until 18 months). It strongly focuses on building rapport with parents. PCH professionals have to attune their care to the unique needs and wishes of each family by taking their point-of-view as basis for the well-child visit, and treat them as equal partners and experts on their child. Professionals aim to further enhance parents’ confidence and parenting skills by motivational and empowering communication. Furthermore, FCC-JointStart provides a guideline for conversation with parents on five domains associated with children’s social-emotional development, i.e. Competence of the primary caretaker; Role of the partner; Social support; Perceived barriers and life events in the context of the child; and Wellbeing of the child (see Appendix 1).

For each domain, professionals can register in the child’s medical record *not discussed*, *protective*, *indistinct*, or a *risk*, with details if needed. After assessment of all domains, PCH professionals jointly decide with parents to rate the situation as “*fine*,” “*not optimal*” indicating that no additional care is needed currently, or “*a problem*” i.e. an additional service needs to be provided by PCH. The FFC-JointStart group had 15 minutes extra for the well-child visit at two months, but not for any other contact. All contacts in the CAU group and all other contact in the FCC JointStart groups lasted 15 minutes.

Before using FCC-JointStart, PCH professionals participated in four days of training by a certified trainer. The training regarded background information on FCC, work instructions, role-play sessions, and discussion of case-vignettes. Within one month after training, PCH professionals had to provide two videotaped well-child visits to the trainer, who assessed the quality of the service delivery using standardized guidelines (with questions like whether all parts of the FCC were discussed and whether PCH-professionals used empowering communication).[21] This procedure was repeated until performance was rated as adequate by the certified trainer. Participating PCH professionals attended supervisory sessions every three months, in which a recording of one of their well-child assessments was assessed based on the afore-mentioned criteria. These supervisory sessions lasted two hours and involved four to six PCH professionals.

### Care-as-usual (CAU) group

In the CAU group, PCH professionals monitored children’s general health and social-emotional development during routine well-child visits according to the guidelines of the National Center for Child Health.[22] These guidelines pay attention to PCH professionals’ communication skills and the importance of children’s development context. In the CAU group, professionals were not trained in FCC components.

### Procedures

At each well-child visit (child ages 2, 3, 4, 6, 7.5, 9, 11, 14, and 18 months), PCH professionals in both groups assessed whether they identified new social-emotional problems or risk factors for developing these and rated the findings as “*fine*,” “*not optimal*,” or “*a problem*”. Missing ratings were substituted by those of the subsequent visit, except for cases with recorded changes in between these visits.

Participants with a recorded need for additional care were invited to fill out additional questionnaires regarding the child’s social-emotional development and developmental context (see S1 Appendix). In the FCC-JointStart group, this involved 114 parents (3.8% of total), out of which 87 (76% of those asked) agreed. In the CAU group, this involved 71 parents (2.6% of total) out of which 60 (86% of those asked) agreed.

One week before the child reached the age of 18 months, all participants received a Child Behavior Checklist (CBCL) 1.5–5,[23,24] at their e-mail address, if available, or by paper, with the request to fill in the questionnaire. If parents did not return the questionnaire within two weeks, they received a reminder, and, after two weeks, parents were contacted by phone. After three phone calls, they received a printed version. 4358 parents returned the questionnaire (response rate 80%), 42 of which were not used because of too many missing responses. All participants received a small gift for their participation.

### Blinding

Professionals could not be blinded of their use of FCC-JointStart, but professional contact regarding the intervention was avoided between professionals from the FCC-JointStart and CAU groups. Parents in the FCC group were made aware that they received a newly developed type of care, but did not know the nature of the evaluation. Parents in the CAU group were informed that they participated in the evaluation of a new type of care, without further details regarding the type of care.

### Measures

The primary outcome was the identification of psychosocial problems and risks for them. This primary outcome was measured by PCH professionals and was rated as “*not optimal*” or “*a problem”*. For both the FCC an the CAU groups, PCH professionals were instructed to rate the situation as “*fine*” if no intervention was needed; as “*not optimal*” if there were some concerns, but no intervention was needed; and as “*a problem*” if an additional activity was needed aimed at the social-emotional wellbeing of the child, like an additional appointment or a referral to a child psychologist. For the FCC-JointStart group, these ratings are likely to be based on a joint decision with parent, since this is an important feature of FCC-JointStart. In the CAU group ratings are likely to be based solely on these criteria; in CAU no extra instruction was given to come to a joint conclusion with parents.

The second primary outcome was the parent-assessed psychosocial development of their child by the Dutch version of the well-validated CBCL 1.5–5.[23,24] The CBCL 1.5–5 consists of 99 problem items which are scored as 0 (not true), 1 (somewhat or sometimes true), or 2 (very true or often true), and can be used to compute Internalizing, Externalizing, and Total problems scores.

The additional questionnaire for participants receiving additional care included a series of measurements on the five domains associated with children’s social-emotional development that were covered by the FCC-JointStart guideline for conversation with parents. These were chosen to optimally assess these domains (see S1 Appendix).

We assessed the following background characteristics: *parental age*, *educational level*, *employment status*, and *country of birth*, and furthermore the *family composition*, having *one or more children*, *birth weight* and *weeks of gestation*. We obtained this information from the child’s medical record, with missing data derived from the baseline questionnaire. Educational level represents the highest level obtained by one of the parents and was divided into low (primary school or less, lower vocational or lower general secondary education), medium (intermediate vocational education, intermediate or higher secondary education) and high (higher vocational education or university).

### Sample size

We determined the sample size based on an improvement of the predictive value of PCH by 20%, with a power of 80% and an alpha of .05.[20] For that we would need 85 “cases” in both regions. Based on a 10% cumulative incidence of problems until age 18 months, 70% of parents agreeing to participate and out of these 70% providing complete cases, we would need 1,750 participants in each of both regions.[20]

### Analyses

First, we described baseline characteristics of the FCC-JointStart and CAU groups, and assessed differences by using Chi-square tests. Second, we compared FCC-JointStart and CAU regarding the rates of first time identified (risks for) social-emotional problems using multilevel logistic regression. In these analysis, we used multilevel analyses to account for a clustering per team. We also adjusted these analyses for potential confounding variables (as listed in Table 2). Third, we compared FCC-JointStart and CAU regarding the degree of early identification, by performing multilevel Kaplan-Meier survival analyses on the time to identification. Fourth, for those participants for whom PCH professionals provided additional care, we assessed the severity of the detected cases based on the measurements regarding the five FCC-JointStart domains. We compared groups using independent t-tests or, in the case of skewed data, Mann-Whitney tests, and computed effect sizes in case of statistically significant differences with small effects being 0.10 to 0.30, and medium effects 0.30 to 0.50.

**Table 2:** Characteristics of participants in the Family-centered care approach (FCC-JointStart) and Care-as-usual group.

	FCC JointStart (n = 2920)	Care-as-usual (n = 2631)	*P* value
**Child’s gender** (male)	1466 (50.2%)	1382 (52.5%)	.084
**Highest education level of the parents**			
Lower	125 (4.8%)	88 (3.6%)	< .001
Secondary	1138 (43.3%)	802 (32.9%)	
Higher	1366 (51.9%)	1547 (63.5%)	
**Age of mother**			
< 20	16 (0.6%)	15 (0.7%)	.801
20 –< 40	2420 (96.8%)	2223 (97.1%)	
40 and over	63 (2.5%)	51 (2.2%)	
**Age of father**			
< 20	5 (0.2%)	6 (0.3%)	.356
20 –< 40	2151 (89.3%)	1987 (90.5%)	
40 and over	252 (10.5%)	202 (9.2%)	
**Employment status (**at least one parent works)	1247 (94.3%)	1430 (94.8%)	.557
**Country of birth (**at least one parent born in the Netherlands)	2534 (99.3%)	2423 (99.1%)	.542
**Family composition** (both biological parents, or biological parent and partner)	2100 (96.6%)	2020 (97.7%)	.042
**Number of children** (one child)	1253 (42.9%)	1084 (41.2%)	.198
**Birth weight (**<2500 grams)	103 (3.9%)	78 (3.5%)	.440
**Gestational age** (<37 weeks)	150 (6.0%)	110 (5.2%)	.258

Finally, we compared the FCC-JointStart and CAU group regarding children’s socio-emotional problems at age 18 months, by analyzing CBCL scores (total, externalizing and internalizing problems scores), crude and adjusted for potential confounding variables as listed in Table 2, using ordinary linear regression analyses, as no clustering per team was found. We repeated these analyses using logistic regression on dichotomized CBCL outcomes (clinical vs. other scores) for all children, and for children for whom PCH professionals had assessed the situation during any of the well-child visits from 2–18 months as being “*not optimal*” or “*a problem*”.

Analyses were done using SPSS20 and SAS 9.4, the cut-off for statistical significance was set at p < .05. Outcomes in analyses were restricted to first identifications per child.

## Results

### Background characteristics

Table 2 shows participants’ baseline characteristics, by assigned group. In the FCC-JointStart group, parents had a slightly lower educational level, and children lived somewhat less frequently in a two-parent family, as compared to the CAU group. Differences were small (Cramer’s V 0.12 and 0.03).

### Earlier and more frequent identification of social-emotional problems

**Fig 2:**
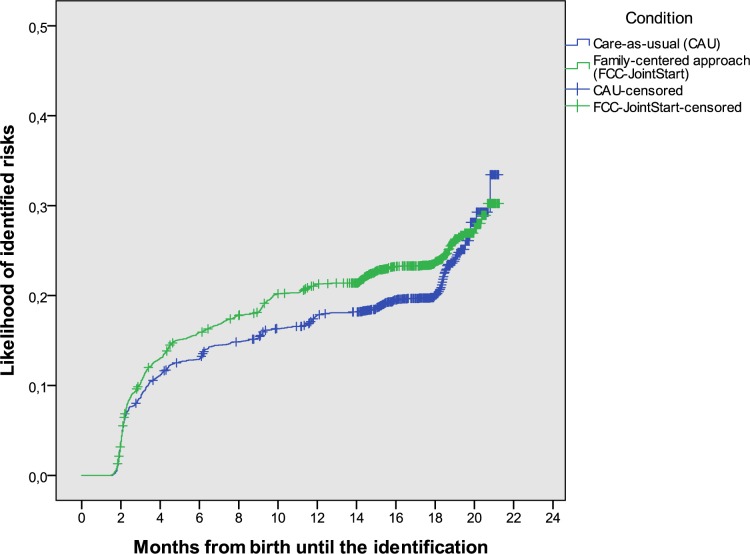
The likelihood of identification of (risks for) social-emotional problems over time, for children receiving family-centered care (FCC-JointStart) or Care-as-usual (CAU).

The rates of newly identified risks for social-emotional problems were higher but not statistically significant, in the FCC-JointStart than in the CAU group (24.7% vs. 22.0%, crude odds ratio, OR (95%-confidence interval, CI) 1.44 (0.96; 2.18), *p =* .02; the effect was small (Phi 0.03). It became larger and statistically significant when adjusted for potential confounding variables (OR 1.94; 95%-CI (1.10; 3.41)). The child age at identification was lower in in the FCC-JointStart group than in the CAU group, see Table 3, with median ages of identification being 108 days in the FFC-JointStart group and 117 days in the CAU group. This was reflected by the Kaplan-Meier survival analysis (Fig 2). Adjusted for clustering, the hazard ratio of earlier identification was 1.18 (1.05; 1.32), confirming a higher likelihood of early identification in the FCC-JointStart group.’

**Table 3:** Overview of the earliest assessment rated as “*not optimal*” or “*a problem*” per child, as compared to all children participating, in the Family-centered care approach (FCC-JointStart) and Care-as-usual group.

Earliest assessment rated as “*not optimal*” or “*a problem*”	FCC JointStart	Care-as-usual
2 months	284 (9.6%)	211 (7.9%)
3 months	93 (3.1%)	76 (2.8%)
4 months	70 (2.4%)	59 (2.2%)
6 months	53 (1.8%)	43 (1.6%)
7.5 months	35 (1.2%)	17 (0.6%)
9 months	66 (2.2%)	39 (1.5%)
11 months	32 (1.1%)	41 (1.5%)
14 months	55 (1.9%)	41 (1.5%)
18 months	46 (1.6%)	65 (2.4%)
Mean age (days)	183	204
Median age (days)	108	117

PCH professionals in the FCC-JointStart group identified more severe cases than in the CAU group, for 6 out of the 15 outcomes (see S1 Appendix). Effect sizes *r* ranged from 0.17 to 0.22 (i.e. all small).

### Psychosocial problems at age 18 months

At age 18 months, the FCC-JointStart and CAU groups did not differ regarding psychosocial problems. Mean CBCL Total Problems scores were 21.4 in the FCC-JointStart group (N = 2199) and 20.8 in the CAU group (N = 2117), *p* = .11. We also did not find statistically significant differences for the Internalizing and Externalizing scores, crude and adjusted. Furthermore, findings for a dichotomized CBCL (clinical vs. other scores) did not differ significantly between FCC-JointStart and CAU (not shown).

## Discussion

To our knowledge this is the first study that has assessed the effectiveness of FCC aiming to improve the early identification of social-emotional problems. We found that an FCC approach (FCC-JointStart) may contribute to more and earlier identification of risks for social-emotional problems, and of families that needed additional care, but not to fewer child psychosocial problems at age 18 months.

Identification of children at risk was more frequent and earlier in the FCC-JointStart group than in the CAU group. A somewhat similar study compared trained to non-trained PCH professionals regarding the identification of psychosocial problems in early school-age children (5–6 years).[25] It found that trained professionals, who used a structured method to assess psychosocial problems, identified moderate and severe problems more accurately than non-trained professionals.[25] The most likely explanation for our finding of earlier and more identification of problems is that the core components of FCC may add to the identification of risks. Potentially, a structured approach may add to that, and should thus be seriously considered in any FCC approach in well-child care.

We found more children at risk with an at average greater risk, and identified them earlier. We previously showed that those identified by the FCC JointStart approach had more severe problems on 6 out of the 15 developmental domains and a similar problem severity on the other domains, compared to CAU. [18] This suggests that early identification was more accurate and, therefore, interventions were provided to families who actually needed it. This may be due to the extensive training of professionals in working with FCC-JointStart. It also suggests that FCC-JointStart may empower parents in such a way that they can handle problems themselves, causing only the more severe cases to still require additional care.

At 18 months of age, we found no differences between the FCC-JointStart and CAU group regarding children’s psychosocial problems, which contrasts with the earlier identification of more and more severe risks for social-emotional problems. An effective handling of these problems might be expected to lead to a reduction of the level of problems at 18 months in the FCC-JointStart group as problems would have been treated earlier and more extensively. However, findings on the CBCL at 18 months did not show this. Reasons may be that the CBCL is not sufficiently sensitive to detect these problems at this age, that attrition was selectively different between the two conditions, or that FCC-JointStart has no effect. Regarding the first potential reason, previous research has shown that the CBCL focusses on relatively severe problems which may not fully reflect the domain that is addressed by well-child care.[26] Regarding the second explanation, we did not find major differences in loss to follow-up which might lead to such a selective attrition. This definitely deserves additional study with other outcome measures and a longer follow-up.

### Strengths and limitations

Major strengths of our study are the inclusion of a large group of children with a rather long follow-up in routine well-child care and a small loss to follow up, in a well-powered quasi-experimental design. Moreover, our study concerned routine well-child care and full communities, which highly adds to the generalizability of the findings. However, our study also has some limitations. First, background characteristics of the two groups differed somewhat, as well as participation rates. These differences were small and were adjusted for in the analyses, but unmeasured factors may still have affected our findings though a significant impact is unlikely given the large differences that we found. Second, we had no golden standards for the appropriateness of risk identification, but we used the best available valid proxies for this. Third, PCH professionals in the CAU group may have had some knowledge about family-centered care, for example through the Internet. If so, this may have led to an underestimation of the effectiveness of FCC-JointStart, but effects are probably small as we avoided any publicity on this project. Fourth, PCH professionals in the FCC and CAU groups may have differed in performance in advance of the study. This is rather unlikely given the very uniform training of PCH professionals, the similarity of the groups of participating professionals in both regions, and the highly standardized way of working, but evidently we cannot exclude this fully. Finally, PCH professionals in the FCC and CAU groups may have labeled the severity of problems in a different way. This may have been in part an intervention outcome as aimed for. The simultaneous increase of the number and the severity of identified problems suggests that this indeed may lead to a better identification and not just to the labeling of more children as having problems.

## Conclusion

FCC-JointStart may contribute to the identification of more risks at an earlier age, with relatively small effects which, however, apply to all children. Therefore, potential population benefits are rather large. Furthermore, FCC-JointStart also seems to be associated with a better identification of risks for socio-emotional problems and problems that need additional care. Further research is needed to assess whether early identification and intervention improve child health outcomes in the long-term.

## Appendices

### Appendix 1 Overview of the contents of the family-centered approach; its five domains and corresponding questions

1. **Competence of the primary caretaker**
  - How do you like being a mother (of … children)?
  - Does the situation correspond to what you expected?
  - Do you feel uncertain or do you have any difficulties with certain aspects of care? If you have, what kind of aspects are these?
  - To what extent do you have time for yourself or for other activities?
  - How do you think your health is?
  *Summarizing: the competence of the parent can be concluded as*…
2. **Role of the partner**
  - How does your partner feel about having a child?
  - To what extent does your partner contribute to the care of your child?
  - To what extent are you satisfied with the contribution of your partner?
  - To what extent do you and your partner agree on how to raise and care for children?
  - What happens if you and your partner do not agree (about how to raise and care for children)?
  - How is the relationship between you and your partner in general?
  (in case of no relationship: how do you feel about that?)
  - What is the impact of having a child on your relationship?
  *Summarizing: the role of the partner can be concluded as*…
3. **Social support**
  - Who supports you emotionally in caring for your child?
  - Who supports you in practical terms in caring for your child?
  - Who advises you about caring for your child?
  - To what extent do you manage with the support you receive?
  - Are you familiar with ways to enlarge your social network?
  - To what extent are you in need of contact with other mothers with babies?
  - How would you define your relationship with your own parents?
  *Summarizing: the social support can be concluded as*…
4. **Perceived barriers and life events in the care-giving context of the child**
  - Have there been any life events the past year?
  If so: To what extent does this influence your contact with (name of the child)?
  - How does the combination of work and child care services work for you?
  - How is your financial situation?
  - How is your housing situation?
  - Are there any other circumstances that impact on your family?
  *Summarizing: the perceived barriers and life events within the care-giving context can be concluded as*…
5. **Wellbeing of the child**
  - How is (name of the child) doing overall?
  - How is (name of the child) developing on a social-emotional level according to you?
  - How familiar are you with (name of the child)?
  - How does (name of the child) respond to his/her environment?
  - To what extent do you recognize different ways of crying?
  *Summarizing: the wellbeing of the child can be concluded as*…

## Supporting information

S1 Appendix Questionnaires regarding the various domains of the family-centered approach. (DOCX)

S2 Appendix CONSORT checklist regarding the manuscript. (DOCX)

S3 Appendix Design paper of the study (Hielkema M, de Winter AF, de Meer G, Reijneveld SA. Effectiveness of a family-centered method for the early identification of social-emotional and behavioral problems in children: a quasi experimental study. BMC Public Health. 2011; 11: 63).
(PDF)

S4 Appendix Research protocol as submitted to the Medical Ethics Committee of the University Medical Center Groningen [in Dutch]. (DOC)

## Abbreviations

CAU: Care-as-usual
CBCL: Child Behavior Checklist
FCC: Family-centered care
PCH: Preventive Child Healthcare
