# Supplementary material for: The added value of a family-centered approach to optimize infants’ social-emotional development: A quasi-experimental study
Source: PLoS One. 2017 Dec 21;12(12):e0187750. doi: 10.1371/journal.pone.0187750 (PMC5739404; doi:10.1371/journal.pone.0187750)
Supplement: S1 Appendix — (DOCX) [file pone.0187750.s001.docx]

**S1 Appendix Questionnaires regarding the various domains of the family-centered approach**

| **Domain of the Family-centered approach** | **Criterion (ES)** | **Nr. of items** | **Measuring** | **Cronbach’s alpha** | **References** |
| --- | --- | --- | --- | --- | --- |
| **Wellbeing of the child** | Ages and Stages Questionnaire Social Emotional (*ASQ-SE*) (versions 6, 12, and 18 months) | 22-29 | Social-emotional development of the child | 0.59-0.78 | [27] |
| **Competence of the parent** | Dutch Parenting Stress Index (*PSI*) (4 subscales) | 11 | Parental competence and attachment | 0.83 | [28] |
|  | Parenting Tasks Checklist or Problem Setting and Behavior Checklist *(PSBC)(Setting Self-Efficacy subscale;* 0.17)* | 14 | Perceived ability of the primary caretaker in mastering problem situations | 0.87 | [29] |
|  | Parental Sense of Competence scale (*PSOC*) | 16 | Competence of the parent | 0.85 | [30-32] |
|  | SF-12 Health Survey  *SF-12 mental* (0.21)*  *SF-12 physical* | 12 | Health status (physical and mental) of the parent | 0.68  0.70 | [33-35] |
| **Role of the partner** | McMaster Family Assessment Device (*FAD*) (General Functioning subscale; 0.22)* | 12 | Emotional relationships within families | 0.94 | [36,37] |
|  | Dutch Parental Stress Index (*PSI*) (subscale partner) | 5 | Having a child and its effect on the relationship between partners | 0.68 | [28] |
| **Social support** | Social Support List, short version (*SSL*)  *Received*  *Shortage* | 12 | Social support | 0.73  0.79 | [38] |
|  | Loneliness score (0.20)*  Social Emotional (0.19)* | 11 | Overall feelings of emotional and social loneliness | 0.84  0.85 | [39] |
| **Perceived barriers or life events within the care giving context of the child** | Questionnaire on the material or social deprivation of a child due to lack of money (*deprivation questionnaire)* | 15 | The material or social deprivation of a child due to shortage of money | 0.69 | [40] |
|  | Dutch Parental Stress Index (*PSI* )(subscale life events; 0.21)* | 17 | Life events that happened in the past year | not applicable | [28] |

**Questionnaires on which statistically significantly higher scores (i.e. worse outcomes) were found for cases that received additional care in the FCC group as compared to in the CAU group; effect sizes between brackets*

**References**

27. Squires J, Bricker D, Heo K, Twombly E. Identification of social-emotional problems in young children using a parent-completed screening measure. Early Childhood Research Quarterly. 2001;16: 405-419.

28. Brock de AJLL, Vermulst AA, Gerris JRM, Abidin RR. PSI Dutch, NOSI-Nijmegen Parenting Stress Index, Manual experimental version [in Dutch]. Lisse: Swets en Zeitlinger. 1992.

29. Sanders MR, Woolley ML. The relationship between maternal self-efficacy and parenting practices: Implications for parent training. Child: Care, Health and Development. 2005;31: 65-73.

30. Lovejoy CM, Verda MR, Hays CE. Convergent and discriminant Validity of Measures of parenting efficacy and control. Journal of Clinical Child Psychology. 1997;26: 366-376.

31. Ohan J, L., Leung DW, Johnston C. The parenting sense of competence scale: evidence of a stable factor structure and validity. Canadian Journal of Behavioural Science. 2000;32: 251-261.

32. Gilmore L, Cuskelly M. Factor structure of the Parenting Sense of Competence scale using a normative sample. Child: Care, Health and Development. 2009;35: 48-55.

33. Jenkinson C, Layte R. Development and testing of the UK SF-12 (short form health survey). J Health Serv Res Policy. 1997;2: 14-18.

34. Jenkinson C, Layte R, Jenkinson D, Lawrence K, Petersen S, Paice C, et al. A shorter form health survey: can the SF-12 replicate results from the SF-36 in longitudinal studies? J Public Health Med. 1997;19: 179-186.

35. Gandek B, Ware JE, Aaronson NK, Apolone G, Bjorner JB, Brazier JE, et al. Cross-validation of item selection and scoring for the SF-12 Health Survey in nine countries: results from the IQOLA Project. International Quality of Life Assessment. J Clin Epidemiol. 1998;51: 1171-1178.

36. Byles J, Byrne C, Boyle MH, Offord DR. Ontario Child Health Study: Reliability and validity of the General Functioning subscale of the McMaster Family Assessment Device. Fam Process. 1988;27: 97-104.

37. Wenniger WF, Hageman WJ, Arrindell WA. Cross-national validity of dimensions of family functioning: First experiences with the Dutch version of the McMaster Family Assessment Device (FAD). Personality and Individual Differences. 1993;14: 769-781.

38. Sonderen van E. Sociale Steun Lijst - Interacties en sociale Steun Lijst - Discrepanties, een handleiding. [Social support list - Interactions and Social Support List - Discrepancies, a manual.]. Groningen: University of Groningen, Northern Centre for Health Care Research. 1993.

39. Jong Gierveld de J, Tilburg van T. Manual of the Loneliness Scale. 2011. Available: <http://home.fsw.vu.nl/TG.van.Tilburg/manual_loneliness_scale_1999.html>. Accessed September 10 2017.

40. Rots-de Vries de C. Rich evidence for poor families exploring the potential of pragmatic-driven intervention research in Preventive Child Healthcare. Tilburg: Tilburg University, 2010.
