# Supplementary material for: The added value of a family-centered approach to optimize infants’ social-emotional development: A quasi-experimental study
Source: PLoS One. 2017 Dec 21;12(12):e0187750. doi: 10.1371/journal.pone.0187750 (PMC5739404; doi:10.1371/journal.pone.0187750)
Supplement: S3 Appendix — Effectiveness of a family-centered method for the early identification of social-emotional and behavioral problems in children: a quasi experimental study. BMC Public Health. 2011; 11: 63). (PDF) [file pone.0187750.s003.pdf]

Hielkema et al. BMC Public Health 2011, 11:636
http://www.biomedcentral.com/1471-2458/11/636

BMC Public Health

STUDY PROTOCOL | Open Access

Effectiveness of a family-centered method for the early identification of social-emotional and behavioral problems in children: a quasi experimental study

Margriet Hielkema[1*], Andrea F de Winter[1], Gea de Meer[2] and Sijmen A Reijneveld[1]

Abstract

Background: Social-emotional and behavioral problems are common in childhood. Early identification of these is important as it can lead to interventions which may improve the child's prognosis. In Dutch Preventive Child Healthcare (PCH), a new family-centered method has been implemented to identify these problems in early childhood. Its main features are consideration of the child's developmental context and empowerment of parents to enhance the developmental context.

Methods/design: In a quasi-experimental study, embedded in routine PCH in the Netherlands, regions in which the family-centered method has been implemented (intervention condition) will be compared to "care as usual" regions (control condition). These regions are comparable in regard to socio-demographic characteristics. From more than 3,500 newborn babies, 18-month follow-up data on social-emotional and behavioral development will be obtained. PCH professionals will assess development during each routine well-child visit; participating parents will fill in standardized questionnaires.

Primary outcomes in the study are the proportion of social-emotional and behavioral problems identified by PCH professionals in children aged 2-14 and 18 months in both conditions, and the proportion of agreement between the assessment of PCH professionals and parents. In addition, the added value of the family-centered approach will be assessed by comparing PCH findings with standardized questionnaires. The secondary outcomes are the degree to which the needs of parents are met and the degree to which they are willing to disclose concerns.

Discussion: The family-centered method seems promising for early identification of social-emotional and behavioral problems. The results of this study will contribute to evidence-based public health.

Trial registration: NTR2681

Background

Behavioral and social-emotional problems are common in childhood and may interfere severely with the everyday life of the child and his/her family [1,2]. Prevalence rates differ between studies and informants, with estimates ranging from 7% to 24% for children under 3 years of age [1,3-5]. For children aged 1 year, Briggs-Gowan et al. found that parents reported problems for approximately 6% of all children [1].

Early identification of social-emotional and behavioral problems, henceforth referred to as psychosocial problems, is important as it can lead to early intervention. Early intervention may help to optimize the environment of the child. This in turn may promote the development of the child [6-8], since the young brain is rapidly developing under the influence of both genes and experience [9-12].

Identification of psychosocial problems in young children is a difficult process, however. In infancy rapid

* Correspondence: m.hielkema@umcg.nl
[1]Department of Health Sciences, University Medical Center Groningen, University of Groningen, Antonius Deusinglaan 1, 9713 AV Groningen, the Netherlands
Full list of author information is available at the end of the article

BioMed Central

© 2011 Hielkema et al; licensee BioMed Central Ltd. This is an Open Access article distributed under the terms of the Creative Commons Attribution License (http://creativecommons.org/licenses/by/2.0), which permits unrestricted use, distribution, and reproduction in any medium, provided the original work is properly cited.

developmental changes occur, along with behaviors which may seem deviant in older children but which can be part of normal development at younger ages [2]. Moreover, development of children is always embedded within a larger context, in which different factors such as, for example, characteristics of both parents and the child, constantly interact and influence each other, as reflected in the bio-ecological model of Bronfenbrenner [2,13]. Different factors may influence the development of children both in a positive or negative way, respectively labeled as protective factors; one example is adequate parenting, along with risk factors, such as lack of support. The influence of both risk and protective factors cannot be evaluated separately from each other; the balance between the burden experienced by parents, and the capacity and resources of the parents should always be evaluated.

The identification process is not only complex but also delicate. Ringing alarm bells too early can cause unnecessary stress, concern, and possible stigma for the parents. But when rung too late, parents may feel misunderstood, may lose trust in the care, their feelings of self-efficacy may decline, and problems may worsen [2]. To identify psychosocial problems or risk factors which may negatively influence psychosocial development, disclosure of any possible concerns by the parents is an important requisite [14-16]. Parental concerns have been described as being as accurate as quality screening instruments are [14]. Factors related to disclosure are: asking questions about psychosocial issues, expressions of support, and listening on the part of professionals [17].

Recently, a family-centered method, in which the above-mentioned difficulties, delicacies, and requisites are kept in mind, was introduced into Preventive Child Healthcare (PCH) in the Netherlands. PCH occupies a unique position in which to monitor psychosocial development closely, comparable to community pediatrics in the USA. Monitoring health and identification of psychosocial problems are mandatory tasks of PCH. PCH is free of charge regardless of insurance situation, and more than 90% of all children are seen regularly during routine well-child visits offered by Child Health Professionals, that is, nurses and doctors, henceforth referred to as CHPs.

As its name implies, the new approach is family-centered, which can be described as "placing the needs of the child, in the context of their family and community, at the centre of care and devising an individualized and dynamic model of care in collaboration with the child and family that will best meet these needs" [18]. The contents of the family-centered approach are based on the bio-ecological model of Bronfenbrenner [13] which reflects different child and contextual characteristics, and the interaction between these, influencing the development of the child. The model has been described as a promising framework for providing support to children in a successful way that is integrated into community-based services [19]. In the family-centered approach, the bio-ecological model is reflected in five different domains which are to be discussed with parents during each routine well-child visit and which concern the broad developmental context of the child. In addition to its contents, the family-centered approach is aimed at building a trusting and supportive relationship with parents in order to stimulate disclosure by and empowerment of the parents, and thus to enhance the positive psychosocial development of the child.

The family-centered approach seems to be a promising method for accurately monitoring psychosocial development, and the context in which infants grow up, in a way that enhances psychosocial development and early intervention if needed. In earlier research by Tan [20], internal validity and reliability of the family-centered approach were rated satisfactory. Furthermore, it was assessed that some domains of the family-centered approach showed a medium-significant correlation with the stress experienced by parents and family needs. The predictive value of the family-centered approach for identification of (risks for) social-emotional problems, along with the external validity of the five domains separately, were not studied by Tan, and is therefore still unknown.

The aim of this study is to assess the added value and the effectiveness of the family-centered approach in terms of how well it monitors psychosocial development and those factors which may influence psychosocial development, in infants of 0-18 months in a PCH setting. It is hypothesized that with the family-centered approach, CHPs will be able to identify psychosocial problems better, as compared to care as usual. Furthermore, it is hypothesized that, with the family-centered approach, the predictive values of the identification of psychosocial problems will be more accurate and that care will be better attuned to parents' needs and wishes and that parents will be more willing to disclose concerns, as compared to care as usual.

Methods/design

Design

In a quasi-experimental design, those regions in which the family-centered approach has already been implemented (intervention condition) will be compared to those regions where care as usual has been maintained (control condition). Overall, the regions in the family-centered care condition and the control condition are comparable for socio-demographic variables, including income, working participation, ethnicity, and percentage

of single-parent households. In Figure 1 the design of the study is described schematically. Randomization per child/family is not possible in this setting as professionals provide care to all children in the region in which they work, in other words, contamination is inescapable in case of individual randomization. We will minimize the likelihood of contamination by prohibiting overlap between CHPs working in both the intervention and control conditions, and by informing CHPs about the activities to be undertaken for data collection in

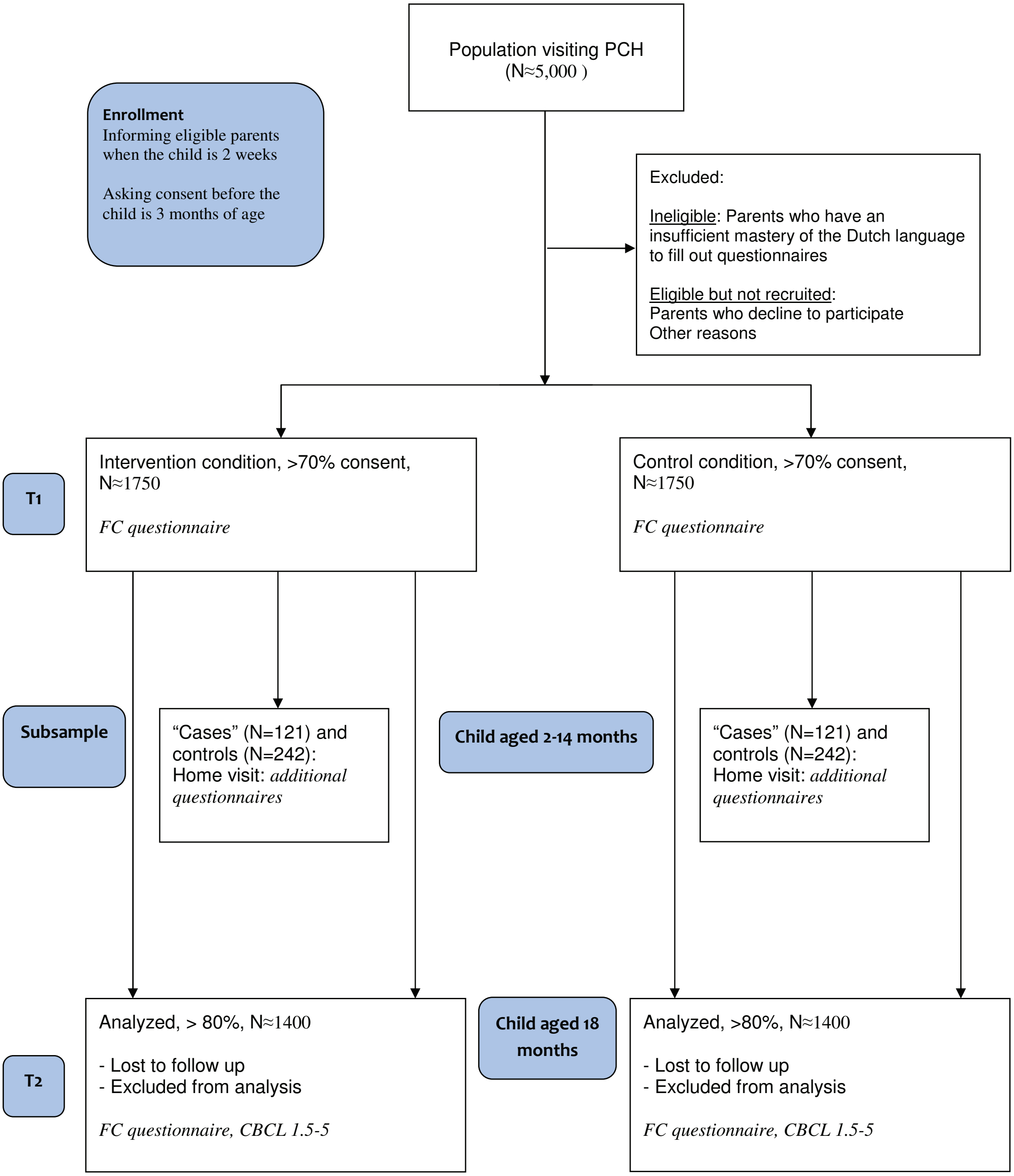

For all participating children aged 0-18 months, CHPs will register within the medical records whether they have identified any (risks for) psychosocial problems during each regular well-child visit.

Figure 1 Study design. This figure describes the design of this study schematically.

both conditions, separately. We chose a quasi-experimental design because full cluster-randomization was not possible due to implementation of the family-centered approach in a number of regions before the study started. To exclude those factors outside the intervention would affect the outcomes; no innovations regarding the psychosocial development of children aged 0-18 will be implemented in either the intervention or the control regions.

The study has been approved by the Medical Ethics Committee of the University Medical Center Groningen. Participation is voluntary and all participants will be asked to give their informed signed consent. The CONSORT statement has been followed in describing the study [21].

Participants

Consecutively, parents of all newborn babies, visiting a large Dutch PCH organization in a number of regions in the north of the Netherlands (parts of the provinces of Drenthe and of Overijssel), will be recruited for participation. Parents are eligible for participation if they visit a PCH center with their newborn before the child reaches 3 months of age and if they have sufficient mastery of the Dutch language to fill out the questionnaires used in the study.

Training

Before the study began, we trained all CHPs for half a day. In the training we provided background information on the study and focused on the inclusion procedure, data collection, and enrolling "cases" in the study. Separate training sessions were held for CHPs from the control and intervention regions.

Procedure

At the time of the routine PCH postnatal home visit, all trained CHPs will inform parents of children aged 2 weeks of their eligibility. The PCH nurse will provide an information package, including a letter, an information leaflet containing information about the study and its aims, and a small gift. CHPs will obtain informed consent from parents before the child reaches the age of 3 months and will subsequently send the consent form to the research institute. For parents who indicate that they do not want to participate, the CHP will ask whether the parent would agree to share some background characteristics (age, gender, country of birth, and employment status of the parents) and the assessment by the CHP of the psychosocial wellbeing of the child at 8 weeks of age. After the consent of the participants is received by the research institute, parents will receive a family-centered questionnaire by mail. At the end of the study, when a child is 18 months of age, parents will receive the family-centered questionnaire again and the Child Behavior Checklist (CBCL) 1.5-5 [22,23]. To enhance the filling out of the questionnaires by the parents, we will send reminders two weeks after sending out the questionnaires. Phone calls are planned one week after sending the reminder to those parents who have not yet returned the questionnaire.

During each routine well-child visit when the child is 2, 3, 4, 6, 9, 11, 14, and 18 months of age, CHPs will register in the medical records for all parents participating whether they have identified psychosocial problems or factors which might negatively influence psychosocial development. When an additional activity from the CHP is needed regarding psychosocial development (e.g., an additional appointment to assess the situation more in depth, an intervention, or a referral), that family (then referred to as a "case") will be asked by the CHP to take part in an interview consisting of several standardized questionnaires concerning the family-centered approach domains. If parents agree to participate, a trained interviewer will visit the parents at home to enhance the participation of risk groups. For each "case," two families will be invited for whom no additional activity was performed (control families). Children will be matched by age, gender, and region (intervention or control). All the families who are interviewed together will form the subsample in our study.

To enhance the compliance of all CHPs, we will monitor all the results (such as inclusion percentages and filling in medical records) very closely from the start and will present these during team meetings. To minimize missing data from CHPs, data collection in the medical records will be closely monitored. When CHPs fail to fill in information for a participating child, they will receive an e-mail with the request to fill in the information in retrospect if possible. To minimize parental attrition, all participating children will be sent a birthday card for their first birthday. At the end of the study, when the child is 18 months of age, all participants will receive a small gift.

Intervention: family-centered approach

Before data collection started, all CHPs, that is, nurses and medical doctors (N = 57), from the intervention region attended group training sessions lasting four days in total before working using the family-centered approach. Training sessions consisted of background information on the family-centered approach, work instructions, role-play sessions, and discussing practical cases. After the group training sessions, the CHPs practiced the family-centered approach during routine well-child visits. Within one month after the training sessions, CHPs were asked to videotape two well-child visits which they discussed with, and which were evaluated

by, trainers using standardized guidelines [20]. This procedure was repeated until the trainer and CHP rated the performance of the CHP as adequate. After passing this assessment, intervision groups of CHPs with trainers were held every three months in order to monitor performance.

The family-centered approach covers five domains associated with psychosocial development which are discussed from the perspective of parents. Domains discussed are: Competence of the parent, Role of the partner, Social support, Perceived barriers or life events within the care-giving context, and Wellbeing of the child. For each domain, several questions regarding that specific domain are asked, intertwined in a conversation, by the CHP (see Additional File 1: Appendix 1). During the second well-child visit at age 8 weeks, the nurse is allotted 15 minutes extra (added to the routine 15 minutes, i.e., 30 minutes in total) to discuss the 5 domains exhaustively. During every routine well-child visit, any possible parental concerns will first be elicited which will provide a starting point for further communication. For all the questions in the family-centered approach, CHPs will be able to register important information as not discussed, a protective factor, not known, or a risk factor. Furthermore, for each domain, the results of the conversation will be able to be summarized as not discussed, a protective factor, not known, or a risk factor, and subsequently an explanation will be able to be provided. Based on the information about the different domains, the parent and the CHP will jointly decide whether there are any concerns. If there are any, an additional activity (for example, an appointment to further clarify these or an intervention) will be planned. In communication with the parents, building a relationship of trust and empowerment of the parents are central features of the family-centered approach. Parents are regarded as experts on their child and in their own strengths, which may function as protective factors that can be enhanced to stimulate positive psychosocial development of the child.

Control condition: care as usual

The care as usual provided by CHPs (N = 49) involves examining and monitoring the general health and psychosocial development of children during regular well-child visits of 15 minutes. During the well-child visit, CHPs follow the Guidelines of the Dutch National Centre for Preventive Child Healthcare [24]. This center provides, monitors, and improves on the national guidelines regarding monitoring developments in Dutch PCH (http://www.ncj.nl).

Outcome measurements

There will be several primary outcomes from this study. The first of these will be the proportion of psychosocial problems identified by the CHPs in both the intervention and control regions. When the child is aged 2 to 14 months, the focus will be on social-emotional development, for children of 18 months of age behavioral problems will be taken into account as well. A second primary outcome will be the predictive value of CHPs' identifying psychosocial problems when a child is between 2 and 14 months old, and later at 18 months, in both the intervention and control conditions. The last primary outcome will be the concordance between the risk and protective factors as assessed by CHPs using the family-centered approach domains (Competence of the primary caretaker, Role of the partner, Social support, Perceived barriers or life events within the care giving context of the child, and Wellbeing of the child) and the outcomes on standardized questionnaires filled in by the parents in the subsample regarding these domains.

The secondary outcomes in the study will be the degree to which the needs of the parents are met and their willingness to disclose their concerns.

Measurements

Social-emotional and behavioral development will be assessed by both the CHPs and the parents. CHPs will indicate during each routine well-child visit between the ages of 2 and 18 months whether psychosocial development is fine, not optimal (but no extra care is needed), or whether there is a problem, indicating that an additional activity is needed. The definition of an additional activity is used to assess whether risks for or actual psychosocial problems exist. From 2-14 months, parents in the subsample of the study will assess the social-emotional development of their children by filling in the Ages and Stages Questionnaire Social Emotional (ASQ-SE) [25,26], an internationally validated questionnaire containing 22 to 29 items for children aged 3 to 60 months. When the child is 18 months of age, all participating parents will fill in the Child Behavioral Checklist (CBCL) 1.5-5, an internationally validated instrument containing 100 items that assesses psychosocial problems [22,23].

The competence of the primary caretaker will be assessed by CHPs within the family-centered approach format by registering whether the competence is regarded as a protective factor, unknown, or a risk factor. Parents from the subsample will indicate their competence by answering 11 items in the Dutch Parental Stress Index (PSI) [27]. Furthermore, the Setting Self-efficacy subscale (14 items) of the Problem Setting and Behavior Checklist (PSBC), measuring the confidence of the primary caretaker in mastering problem situations [28], and the Parental Sense of Competence scale (PSOC), 16 items measuring the competence of the

parent [29] will be used. With the SF-12, an abbreviated version of the 36-Item Short Form Health Survey [30,31], the health status (physical and mental) of the parent will be assessed.

The role of the partner will be assessed by CHPs by indicating whether the role can be seen as a protective, unknown, or risk factor. Parents in the subsample of the study will assess the relationship between the partners using the 12-item General Functioning (GF) subscale of the McMaster Family Assessment Device (FAD) that addresses the emotional relationships within families [32,33]. Furthermore, having a baby and the effect on the relationship between the partners will be assessed using the subscale "relationship" of the Dutch Parental Stress Index (5 items) [27].

Social support will be assessed by the CHPs by registering whether this can be perceived as a protective factor, unknown, or a risk factor. In the additional interview of the subsample, parents will indicate their social support by making use of a short version of the Social Support List (SSL, short version) [34], containing 12 items addressing the social support experienced. Furthermore, the Loneliness score, containing 11 items assessing feelings of overall, emotional, and social loneliness [35], will be used.

Perceived barriers or life events within the care-giving context of the child will be assessed by the CHPs by indicating in the family-centered approach format whether these can be seen as a protective factor, unknown, or a risk factor. Parents in the subsample will indicate the barriers they perceive within the care-giving context of the child by using a questionnaire measuring the relationship between basic requirements and potential deprivations for the child (e.g., nutrition) and the financial situation of parents [36]. Furthermore, a list with 17 items of life events which happened in the past year, derived from the Dutch Parental Stress Index [27], will be used.

The met and unmet needs of parents will be assessed using a family-centered questionnaire designed for this study, filled in by all participating parents when the child is 2 and 18 months of age, which assesses the needs and experiences of parents in terms of PCH.

Willingness to disclose will be measured by asking all parents to rate the following statement: "I feel free to discuss all kinds of worries with the PCH professionals" on a Likert scale from 1 (= not true at all) to 5 (= very true) when the child is 2 and 18 months of age.

Other outcome measurements will deal with the background characteristics assessed at baseline, including children's and parents' ages and genders, parental educational level, employment status, country of birth, and length of time living in the Netherlands. In the subsample, possible biological vulnerabilities within the family will also be assessed by asking participants whether there are any family members familiar with different kinds of psychopathology.

Sample size and power calculation

In a study regarding children aged 2-4 years, PCH identified psychosocial problems in 10-12% of all children, of these 22-23% were confirmed by clinical scores on the CBCL filled in by parents [37]. For the current study, an increase in the predictive value of 20% for the family-centered approach is considered to be relevant, resulting in an identification rate of 42%. With a power of 80% and a .05 alpha, 85 "cases" in both regions of the country will be needed to detect a change in predictive value of 20%.

Based on birth statistics in both the intervention and control regions, approximately 2,500 births are expected [38] within one year in both the intervention and control regions. With an expected participation rate of 70%, this would result in 1,750 participating families in both conditions within the inclusion period of one year. With an expected cumulative incidence of 10% of children with social-emotional problems between 2 and 14 months, this would result in 175 "cases" in both conditions. We anticipate that 70% of "cases" will agree to participate, so that 121 "cases" and 242 matching control families can be invited for complementary interviews. For this group, we anticipate that for 70% of included "cases" a complete dataset will be collected.

Time frame

The aim is to have an inclusion period of one year. As it is uncertain whether an identification rate of psychosocial problems of 10% will be feasible when the child is between 2 and 14 months of age, the inclusion period can be spread over a period of 20 months. Consecutively, CHPs will then ask parents who visit the PCH center with their newborns to participate before the child reaches the age of 3 months. When the child reaches the age of 2-14 months, "cases" and matching control families will be enrolled in the subsample. The final measurement for all participating families will take place when the child is 18 months of age, and will be spread over a period equal to the length of inclusion.

Statistical analyses

To compare the baseline characteristics of the participants in the intervention and control regions, chi-squared tests for categorical variables and t-tests for continuous variables will be used. If the intervention and control regions differ regarding the background characteristics of the children, appropriate multivariable analyses will be done using standard and logistic regression analyses to adjust for these differences.

Regarding the primary outcomes of the study, the following analysis will be performed. First, we will compare the proportion of, and risks for psychosocial problems identified by the CHPs in both the intervention and control conditions when the child is between 2 and 14 months of age and when the child is 18 months of age, using chi-squared tests and logistic regression analysis to correct for potential differences between regions. Second, we will assess the sensitivity, specificity, and the positive and negative predictive values of social-emotional and psychosocial problems identified by CHPs in both conditions, using the ASQ-SE [25,26] for children aged 2-14 months from the subsample and using the CBCL [22,23] for all participating children when the child is 18 months of age. Third, we will compare kappas as a measurement of agreement between the protective and risk factors assessed by the CHPs, and relevant reference questionnaires as filled in by the parents from the subgroup.

For the secondary outcomes of the study, we will compare met and unmet needs of the parents between conditions using independent t-tests and multivariate regression analysis to correct for potential differences in background characteristics. The level of willingness to disclose concerns will be compared using ordinal regression analysis.

Data will be analyzed using SPSS 18.0. The significance level is set at .05.

Discussion

This paper presents the design of a quasi-experimental study whose aim is to assess the added value and effectiveness of a new family-centered method designed to monitor psychosocial development and those factors which may influence psychosocial development in early childhood. Daily practice needs an evidence-based method to monitor psychosocial development and identify psychosocial problems at an early age, since this may contribute to early intervention, when needed, and thus to the wellbeing of the child and his/her family [6-8,12]. Internationally, the importance of early identification of psychosocial problems is acknowledged [39], and different questionnaires regarding psychosocial development have been developed and studied such as, for example, the Child Behavior Checklist (CBCL) and the Ages and Stages Questionnaire Social Emotional (ASQ-SE) [2]. However, there are no evidence-based methods, aimed at both the psychosocial development of the child as well as at the contextual risk factors, which can be integrated into routine well-child care, although Bright Futures has been described as promising [40]. The theoretical basis of the family-centered approach represents a promising start in supporting children and families in integrating with community-based services successfully [19], and takes into account both the difficulties and delicacies found in the early identification process. If the family-centered approach proves to be effective, its feasibility in routine care will be high because it has already been implemented successfully in routine care in the intervention regions.

Strengths

We expect the findings of this large prospective quasi-experimental study into the daily practice of PCH to be very useful for practitioners and policymakers. Inclusion and exclusion criteria are set so as to highly resemble routine care in order to obtain generalizable findings. For the same reason, we will be investing a great deal in order to enhance the participation of all parents. For example, before the study started, we were able to focus media attention on the study in order to interest potential participants. Furthermore, in the information packet for parents, a small gift is provided to further spark the interest of the parents, and when we wrote the information flyer we made use of input from the CHPs so as to appeal to parents. For that part of the subsample in which an additional activity is to be carried out by a CHP, the parents will be informed by their own CHP and thereafter will be contacted by an interviewer who will visit the families at home. Interviewers are all well trained and have very good communication skills which should enhance participation of families. To further facilitate the participation of parents, we trained all the CHPs before the study started, interactively informing them how to motivate parents adequately and, if necessary, to remove any barriers felt by parents.

Besides evidence regarding the effectiveness of identifying the risks for psychosocial development, our study will also provide insight into whether parents experience the family-centered approach as truly family-centered. This insight may be very useful for the design of further training sessions for the CHPs. Furthermore, the evidence about whether parents feel free to disclose possible concerns to their CHP may provide interesting and important information. Disclosure by parents seems a sine qua non for the early identification of, and risks for, psychosocial problems. Parental concerns have even been shown to be as accurate as other screening methods such as questionnaires [14].

This study will prospectively monitor the development of a large number of children. Therefore, it will provide a wealth of information about the early development of infants and about factors within the child or those contexts which may influence psychosocial development in the first 18 months of life. With this structured way of monitoring psychosocial development at such an early age, we should gain more insight into the normal developmental pathways of children during the first 18 months.

Potential limitations

This study also has some limitations. First of all, randomization will not be possible, since both the CHPs and parents are bound to their PCH regions. However, we will minimize contamination between regions, for example, through separate training sessions for the CHPs, by actively involving management of both the intervention and control regions, and by avoiding that CHPs work in regions of both the intervention and control condition.

Selection bias may also possibly influence the study's findings. To minimize this, we have taken several measures to promote the participation of all parents. As stated above, all the CHPs were instructed on how to pass information onto parents and how to use effective strategies to remove any barriers to participation, both in the overall study population and in the subsample of parents. For those parents who do not want to participate, the CHP will ask whether the parent would agree to share some background characteristics (age, gender, country of birth, and employment status of parents) and the assessment of the psychosocial wellbeing by the CHP when the child is 8 weeks of age. By collecting this information, comparisons between groups can be made to provide insight in the presence of potential selection bias.

One challenge in this study concerns the large number of participating CHPs who all need to comply with the study protocol. However, this reflects daily practice very well, which highly contributes to the generalizability of our findings. Moreover, to enhance the compliance of all CHPs, from the outset we will monitor all results very closely in terms of inclusion percentages and filling in information in the medical records of participating children. Results will be presented during team meetings. With close monitoring, we should be able to provide interventional action at an early stage if needed.

In interpreting results in terms of the predictive value of the CHPs' identification of the psychosocial development of children, it is important to note that we will be using the ASQ-SE for children younger than 18 months and the CBCL 1.5-5 for children aged 18 months as the "gold standard." We should note, however, that this gold standard does not fully reflect the judgment of the CHPs, which is also based on clinical experience. In an ideal situation, we should also gather information from independent experts in order to have a possibly more objective and informative measurement of psychosocial development. This will not be part of our study due to the large numbers and the time-consuming method that would involve.

Conclusions

The family-centered approach seems to be a promising new method for monitoring and enhancing psychosocial development of young children in PCH centers. Our study is the first to assess the added value and effectiveness of the family-centered approach in a large sample. Using an innovative design, we will assess several dimensions of effectiveness in order to come up with a complete overview of the added value of the family-centered approach. In a broader sense, this study will contribute to evidence-based public health.

Additional material

Additional file 1: Appendix 1. Overview of the contents of the family-centered approach; the five domains and corresponding questions. (PDF file).

Acknowledgements
This study was funded by ZonMw, the Netherlands organization for health research and development.

Author details
[1]Department of Health Sciences, University Medical Center Groningen, University of Groningen, Antonius Deusinglaan 1, 9713 AV Groningen, the Netherlands. [2]Municipal Health Service Fryslân, Harlingertrekweg 58, 8913 HR Leeuwarden, the Netherlands.

Authors' contributions
SAR and GdM designed the study which was approved by the funder ZonMw, the Netherlands organization for health research and development. AFdW wrote the first version of the study protocol, information leaflet, and informed consent form. After comments of the Medical Ethical Committee of the University Medical Center Groningen, MH rewrote the study protocol, information flyer, and informed consent. MH wrote the final manuscript which was discussed, edited, and revised by all the authors. All authors read and approved the final manuscript.

Competing interests
The authors declare that they have no competing interests.

Received: 25 July 2011 Accepted: 9 August 2011
Published: 9 August 2011

References

1. Briggs-Gowan MJ, Carter AS, Skuban EM, Horwitz SM: **Prevalence of social-emotional and behavioral problems in a community sample of 1- and 2-year-old children.** *J Am Acad Child Adolesc Psychiatry* 2001, **40(7)**:811-819.
2. Carter AS, Briggs-Gowan MJ, Davis NO: **Assessment of young children's social-emotional development and psychopathology: recent advances and recommendations for practice.** *J Child Psychol Psychiatry* 2004, **45(1)**:109-134.
3. Velderman M Klein, Crone MR, Wiefferink CH, Reijneveld SA: **Identification and management of psychosocial problems among toddlers by preventive child health care professionals.** *Eur J Public Health* 2010, **20(3)**:332-338.
4. Roberts ERobert, Attkisson C Clifford, Rosenblatt Abram: **Prevalence of Psychopathology Among Children and Adolescents.** *Am J Psychiatry* 1998, **155(6)**:715-725.
5. Skovgaard AM, Olsen EM, Christiansen E, Houmann T, Landorph SL, Jorgensen T, CCC 2000 Study Group: **Predictors (0-10 months) of psychopathology at age 11/2 years - a general population study in The Copenhagen Child Cohort CCC 2000.** *J Child Psychol Psychiatry* 2008, **49(5)**:553-562.
6. Herrod HG: **Do first years really last a lifetime?** *Clin Pediatr (Phila)* 2007, **46(3)**:199-205.
7. Weisz JR, Sandler IN, Durlak JA, Anton BS: **Promoting and protecting youth mental health through evidence-based prevention and treatment.** *Am Psychol* 2005, **60(6)**:628-648.

8. Durlak JA, Wells AM: **Evaluation of indicated preventive intervention (secondary prevention) mental health programs for children and adolescents.** *Am J Community Psychol* 1998, **26(5)**:775-802.
9. Shonkoff JP, Bales SN: **Science does not speak for itself: translating child development research for the public and its policymakers.** *Child Dev* 2011, **82(1)**:17-32.
10. Beauchaine TP, Neuhaus E, Brenner SL, Gatzke-Kopp L: **Ten good reasons to consider biological processes in prevention and intervention research.** *Dev Psychopathol* 2008, **20(3)**:745-774.
11. Schore AN: **Dysregulation of the right brain: a fundamental mechanism of traumatic attachment and the psychopathogenesis of posttraumatic stress disorder.** *Aust N Z J Psychiatry* 2002, **36(1)**:9-30.
12. Dawson G, Ashman SB, Carver LJ: **The role of early experience in shaping behavioral and brain development and its implications for social policy.** *Dev Psychopathol* 2000, **12(4)**:695-712.
13. Bronfenbrenner U, Ceci SJ: **Nature-nurture reconceptualized in developmental perspective: a bioecological model.** *Psychol Rev* 1994, **101(4)**:568-586.
14. Glascoe FP: **Parents' evaluation of developmental status: how well do parents' concerns identify children with behavioral and emotional problems?** *Clin Pediatr (Phila)* 2003, **42(2)**:133-138.
15. Regalado M, Halfon N: **Primary care services promoting optimal child development from birth to age 3 years: review of the literature.** *Arch Pediatr Adolesc Med* 2001, **155(12)**:1311-1322.
16. Ellingson KD, Briggs-Gowan MJ, Carter AS, Horwitz SM: **Parent identification of early emerging child behavior problems: predictors of sharing parental concern with health providers.** *Arch Pediatr Adolesc Med* 2004, **158(8)**:766-772.
17. Wissow LS, Roter DL, Wilson ME: **Pediatrician interview style and mothers' disclosure of psychosocial issues.** *Pediatrics* 1994, **93(2)**:289-295.
18. MacKean GL, Thurston WE, Scott CM: **Bridging the divide between families and health professionals' perspectives on family-centred care.** *Health Expect* 2005, **8(1)**:74-85.
19. Avan BI, Kirkwood BR: **Review of the theoretical frameworks for the study of child development within public health and epidemiology.** *J Epidemiol Community Health* 2010, **64(5)**:388-393.
20. Tan NJ, Boom van den DC, Hermanns JJM: **Protocol ter ondersteuning van de sociaal-emotionele ontwikkeling. Een volgsysteem voor consultatiebureau's (0-4 jarigen). Ontwikkeld in opdracht van DMO.** *Universiteit van Amsterdam* Faculteit der Maatschappij- en Gedragswetenschappen, Amsterdam; 2005.
21. Schulz KF, Altman DG, Moher D: **CONSORT 2010 statement: Updated guidelines for reporting parallel group randomised trials.** *J Pharmacol Pharmacother* 2010, **1(2)**:100-107.
22. Ivanova MY, Achenbach TM, Rescorla LA, Harder VS, Ang RP, Bilenberg N, Bjarnadottir G, Capron C, De Pauw SS, Dias P, Dobrean A, Doepfner M, Duyme M, Eapen V, Erol N, Esmaeili EM, Ezpeleta L, Frigerio A, Goncalves MM, Gudmundsson HS, Jeng SF, Jetishi P, Jusiene R, Kim YA, Kristensen S, Lecannelier F, Leung PW, Liu J, Montirosso R, Oh KJ, *et al*: **Preschool psychopathology reported by parents in 23 societies: testing the seven-syndrome model of the child behavior checklist for ages 1.5-5.** *J Am Acad Child Adolesc Psychiatry* 2010, **49(12)**:1215-1224.
23. Achenbach TM, Rescorla LA: *Manual for the ASEBA Preschool Forms & Profiles* Burlington, VT: University of Vermont, Research Center for Children, Youth & Families; 2000.
24. **www.ncj.nl.** [http://www.ncj.nl/onderwerpen/2/richtlijnen].
25. Squires J, Bricker D, Heo K, Twombly E: **Identification of social-emotional problems in young children using a parent-completed screening measure.** *Early Childhood Research Quarterly* 2001, **16(4)**:405-419.
26. Squires J, Bricker D, Potter L: **Revision of a parent-completed development screening tool: Ages and Stages Questionnaires.** *J Pediatr Psychol* 1997, **22(3)**:313-328.
27. Brock de AJLL, Vermulst AA, Gerris JRM, Abidin RR: *NOSI-Nijmeegse Ouderlijke Stress Index, Handleiding experimentele versie [NOSI-Nijmegen Parenting Stress Index, Manual experimental version]* Lisse: Swets en Zeitlinger; 1992.
28. Sanders RMatthew, Woolley ML: **The relationship between maternal self-efficacy and parenting practices: Implications for parent training.** *Child: Care, Health and Development* 2005, **31(1)**:65-73.
29. Gilmore L, Cuskelly M: **Factor structure of the Parenting Sense of Competence scale using a normative sample.** *Child: Care, Health and Development* 2009, **35(1)**:48-55.
30. Jenkinson C, Layte R: **Development and testing of the UK SF-12 (short form health survey).** *J Health Serv Res Policy* 1997, **2(1)**:14-18.
31. Jenkinson C, Layte R, Jenkinson D, Lawrence K, Petersen S, Paice C, Stradling J: **A shorter form health survey: can the SF-12 replicate results from the SF-36 in longitudinal studies?** *J Public Health Med* 1997, **19(2)**:179-186.
32. Byles J, Byrne C, Boyle HM, Offord RD: **Ontario Child Health Study: Reliability and validity of the General Functioning subscale of the McMaster Family Assessment Device.** *Fam Process* 1988, **27(1)**:97-104.
33. Wenniger FW, Hageman JW, Arrindell AW: **Cross-national validity of dimensions of family functioning: First experiences with the Dutch version of the McMaster Family Assessment Device (FAD).** *Personality and Individual Differences* 1993, **14(6)**:769-781.
34. Sonderen van E: *Sociale Steun Lijst - Interacties en sociale Steun Lijst - Discrepanties, een handleiding* Groningen: University of Groningen, Noordelijk Centrum voor Gezondheidswetenschappen; 1993.
35. [http://home.fsw.vu.nl/TG.van.Tilburg/manual_loneliness_scale_1999.html].
36. Rots-de Vries de C: **Rich evidence for poor families exploring the potential of pragmatic-driven intervention research in Preventive Child Healthcare.** Tilburg University, Arnhem; 2010.
37. Reijneveld SA, Brugman E, Verhulst FC, Verloove-Vanhorick SP: **Identification and management of psychosocial problems among toddlers in Dutch preventive child health care.** *Arch Pediatr Adolesc Med* 2004, **158(8)**:811-817.
38. [http://www.cbs.nl/].
39. Foy JM, American Academy of Pediatrics Task Force on Mental Health: **Enhancing pediatric mental health care: report from the American Academy of Pediatrics Task Force on Mental Health. Introduction.** *Pediatrics* 2010, **125(Suppl 3)**:S69-74.
40. Zimmerman BMHS, Gallagher JRN, Botsko CMA, Ledsky RMBA, Gwinner VMPP: **Assessing the Bright Futures for Infants, Children and Adolescents Initiative.** *Findings from a National Process Evaluation* Washington: Health systems Research, Inc.; 2005.

Pre-publication history

The pre-publication history for this paper can be accessed here: http://www.biomedcentral.com/1471-2458/11/636/prepub

doi:10.1186/1471-2458-11-636
Cite this article as: Hielkema *et al.*: **Effectiveness of a family-centered method for the early identification of social-emotional and behavioral problems in children: a quasi experimental study.** *BMC Public Health* 2011 **11**:636.

Submit your next manuscript to BioMed Central and take full advantage of:

- **Convenient online submission**
- **Thorough peer review**
- **No space constraints or color figure charges**
- **Immediate publication on acceptance**
- **Inclusion in PubMed, CAS, Scopus and Google Scholar**
- **Research which is freely available for redistribution**

Submit your manuscript at
www.biomedcentral.com/submit

BioMed Central
