# Supplementary material for: The added value of a family-centered approach to optimize infants’ social-emotional development: A quasi-experimental study
Source: PLoS One. 2017 Dec 21;12(12):e0187750. doi: 10.1371/journal.pone.0187750 (PMC5739404; doi:10.1371/journal.pone.0187750)
Supplement: S4 Appendix — (DOC) [file pone.0187750.s004.doc]

*Effectiviteit van vroegsignalering binnen het programma Samen Starten*

**(Februari 2009)**

**PROTOCOL TITEL: Effectiviteit van vroegsignalering binnen het programma Samen Starten**

| **Protocol ID** | **Projectnummer 80-82435-98-8042** |
| --- | --- |
| **Short title** | **Effectiviteit van vroegsignalering** |
| **Version** | **1** |
| **Date** | **Februari 2009** |
| **Coordinating investigator/project leader** | ***Dr. A.F. de Winter***  ***Gezondheidswetenschappen, sectie sociale geneeskunde, Gebouw 3217, kamer 609***  ***Postbus 196***  ***9700 AD Groningen***  ***050-3638917***  ***a.f.de.winter@med.umcg.nl*** |
| **Principal investigator(s) (in Dutch: hoofdonderzoeker/uitvoerder)**  ***Multicenter research: per site*** | ***Prof.dr. S.A. Reijneveld Gezondheidswetenschappen, sectie sociale geneeskunde, Gebouw 3217***  ***Postbus 196***  ***9700 AD Groningen*** |
|  |  |
| **Sponsor (in Dutch: verrichter/opdrachtgever)** | ***ZonMw*** |
|  |  |
| **Independent physician(s)** | **Mw J. Eggink-Dik** |
|  |  |
|  |  |
| **Laboratory sites <*if applicable*>** | ***Niet van toepassing*** |
|  |  |
|  |  |
| **Pharmacy <*if applicable*>** | ***Niet van toepassing*** |
|  |  |

**PROTOCOL SIGNATURE SHEET**

| **Name** | **Signature** | **Date** |
| --- | --- | --- |
| **Sponsor or legal representative:**  ***<please include name and function>***  **For non-commercial research,**  **Head of Department:**  ***<include name and function>*** | **N.v.t.** |  |
| **Coordinating Investigator/Project leader/Principal Investigator:**  ***<please include name and function>*** | **Prof.dr. S.A. Reijneveld, hoogleraar sociale geneeskunde, voorzitter van de disciplinegroep Gezondheidswetenschappen van het UMCG** |  |
|  |  |  |

**INHOUDSOPGAVE**

1. INTRODUCTIE [6](#__RefHeading___Toc222718388)

2. DOELSTELLING [9](#__RefHeading___Toc222718389)

3. ONDERZOEKSOPZET [10](#__RefHeading___Toc222718390)

4. STUDIE POPULATIE EN UITNODIGING TOT DEELNAME [13](#__RefHeading___Toc222718391)

4.1 Steekproefgrootte [14](#__RefHeading___Toc222718392)

4.2 Terugtrekking van deelname [15](#__RefHeading___Toc222718393)

4.3 Vroegtijdige beëindiging van de studie [15](#__RefHeading___Toc222718394)

5. METHODE [15](#__RefHeading___Toc222718395)

5.1 Procedures [15](#__RefHeading___Toc222718396)

5.2 Meetinstrumenten [16](#__RefHeading___Toc222718397)

6. STATISTISCHE ANALYSE [18](#__RefHeading___Toc222718398)

7. ETHISCHE ASPECTEN [19](#__RefHeading___Toc222718399)

7.1 Regulation statement [19](#__RefHeading___Toc222718400)

7.2 Werving van deelnemers en informed consent [19](#__RefHeading___Toc222718401)

7.3 Verzekering [19](#__RefHeading___Toc222718402)

7.4 Incentives [19](#__RefHeading___Toc222718403)

8. ADMINISTRATIEVE PROCEDURES [20](#__RefHeading___Toc222718404)

8.1 Documentatie en toegankelijkheid van de data [20](#__RefHeading___Toc222718405)

8.2 Publicaties [20](#__RefHeading___Toc222718406)

9. REFERENTIES [21](#__RefHeading___Toc222718407)

|  |  |
| --- | --- |
|  |  |
|  |  |
|  |  |
|  |  |
|  |  |
|  |  |
|  |  |
|  |  |

Samenvatting

**Achtergrond.** Binnen het programma Samen Starten wordt het DMO-protocol gebruikt, een volgsysteem gericht op signalering van opvoedingssituaties die risico’s opleveren voor de sociaal-emotionele ontwikkeling van het kind. Het DMO-protocol wordt als veelbelovend beschouwd maar de effectiviteit van dit protocol voor signalering van (dreigende) sociaal-emotionele problematiek en hiervoor bedreigende situaties is nog niet vastgesteld.

**Doel.** Vaststellen van de effectiviteit van het DMO-protocol voor het signaleren van sociaal-emotionele problematiek binnen de setting van de jeugdgezondheidszorg (JGZ) voor 0-4 jarigen.

**Studie design.** Het onderzoek betreft een longitudinale, observationele studie waarin validatie plaats vindt van het DMO-protocol. Volgens het DMO-protocol wordt vanaf het eerste contactmoment met de JGZ (postnataal huisbezoek bij 2-3 weken) aandacht besteed aan de brede opvoedingssituatie vanuit het perspectief van de ouder(s). Relevante informatie wordt per consult in samenspraak met de ouder(s) vastgelegd op een gestandaardiseerd formulier. Tijdens het consult van acht weken bespreekt de verpleegkundige alle domeinen van het DMO-protocol, i.e. functioneren/welbevinden van het kind, functioneren/welbevinden van de primaire verzorger/opvoeder, rol van de partner, ervaren sociale steun, en obstakels. Daarna spitst de inventarisatie zich toe per gezin, aansluitend bij eerdere informatie en actueel ontwikkelingsmoment.

Binnen het werkgebied van thuiszorgorganisatie Icare wordt de nieuwe werkwijze gefaseerd geïmplementeerd. De implementatie is zodanig gefaseerd dat twee regio’s ontstaan die vergelijkbaar zijn wat betreft sociaaldemografische kenmerken. In de ene regio is het DMO-protocol geïmplementeerd, verder te noemen als ‘DMO-implementatieregio’. In de andere regio zal de JGZ-professional blijven werken volgens de tot dan toe gangbare methodiek, verder te noemen als de ‘DMO-controleregio’.

In beide regio’s zal de JGZ medewerker tijdens de reguliere bezoeken aan het consultatiebureau, vanaf de geboorte tot de leeftijd van 15 maanden, beoordelen of er sprake is van sociaal-emotionele problematiek of van een risicosituatie hiervoor. Gezinnen die als ‘problematisch’ worden geïdentificeerd, worden uitgenodigd voor aanvullend onderzoek. Bij ieder als ‘problematisch’ gesignaleerd gezin worden twee ‘niet-problematische’ gezinnen uitgenodigd voor aanvullend onderzoek. Hierbij worden ‘problematische’ en ‘niet-problematische’ gezinnen gematcht voor geslacht en leeftijd van het kind en dienen ze uit hetzelfde implementatiegebied te komen, i.e. de ‘DMO-implementatieregio’ dan wel de ‘DMO-controleregio’.

**Studie populatie.** Alle ouders in de ‘DMO-implementatie-regio’ en ‘DMO-controle regio’ worden tijdens het postnatale huisbezoek (als het kind 2 a 3 weken oud is) gevraagd om hun medewerking te verlenen aan het onderzoek. De inclusieperiode bedraagt 12 maanden.

**Interventie.** Dit is niet van toepassing.

**Belangrijke uitkomsten.**  Het aanvullend onderzoek bestaat uit gevalideerde vragenlijsten over de sociaal-emotionele ontwikkeling van het kind en de beleving van door problematische situaties op de domeinen van het DMO-protocol. Op de kindleeftijd van 18 maanden ontvangen alle gezinnen die bij de kindleeftijd van 8 weken werden geïncludeerd de Child Behavioural CheckList (CBCL) voorafgaande aan het reguliere bezoek aan de JGZ.

**Aard en omvang van de belasting en risico’s van deelname.** Er zijn geen risico’s verbonden aan de deelname. De bevindingen van de Jeugdgezondheidszorg uit de dossiers worden verzameld en daarnaast vullen professionals en ouders vragenlijsten in ten behoefte van het onderzoek.

# INTRODUCTIE

| **PROBLEEMSTELLING**  Gedragsproblemen en emotionele problemen komen veel voor op de kinderleeftijd met prevalenties van 16-26% (Egger, 2006)(Reijneveld, 2004). Vroege signalering is relevant omdat veel problematiek in de eerste levensjaren ontstaat (Landry, 2001)(Rutter, 2002) en interventies het meest effectief zijn in een vroeg stadium (Durlak, 1998)(Nelson, 2003). Signalering van een stagnerende ontwikkeling behoort tot de wettelijke basistaken van de jeugdgezondheidszorg, voor 0-4 jarigen uitgevoerd door consultatiebureaus. Deze setting is uitermate geschikt voor preventie gericht op alle gezinnen met jonge kinderen, gezien het zeer hoge bereik (landelijk 90-95%).  De Universiteit van Amsterdam heeft in opdracht van de Dienst Maatschappelijke Ontwikkeling (DMO) van de gemeente Amsterdam een protocol ontwikkeld dat de jeugdgezondheidszorg ondersteunt bij de signalering van stagnerende sociaal-emotionele ontwikkeling van kinderen en risicosituaties daarvoor. Het DMO-protocol wordt gebruikt binnen het programma Samen Starten dat effectieve samenwerking beoogt tussen ketenpartners in de zorg rondom psychosociale problematiek bij jonge kinderen (www.samenstarten.nl). Het DMO-protocol is een volgsysteem gebaseerd op het bio-ecologisch ontwikkelingsmodel (Meyers, 1999)(Booth, 2002)(Bronfenbrenner, 2005). Dit model veronderstelt dat de (neurobiologische en gedragsmatige) ontwikkeling van het kind en de ouder-kindrelatie afhankelijk is van continue transactie tussen biologische en sociale factoren, tussen ‘nature’ and ‘nurture’. Als relevante factoren worden onderkend: aanleg/temperament, prenatale omgeving, sociale steun en stressoren binnen en buiten het gezin, sociaal-culturele factoren, en lokaal maatschappelijke factoren (Ramey & Ramey, 1998). Volgens het DMO-protocol wordt vanaf het eerste contactmoment met de |
| --- |
| JGZ (postnataal huisbezoek bij 2-3 weken) aandacht besteedt aan de brede opvoedingssituatie vanuit het perspectief van de ouder(s). Relevante informatie wordt per consult in samenspraak met de ouder(s) vastgelegd op een gestandaardiseerd formulier. Tijdens het consult van acht weken bespreekt de verpleegkundige alle domeinen van het DMO-protocol, i.e. functioneren/welbevinden van het kind, functioneren/welbevinden van de primaire verzorger/opvoeder, rol van de partner, ervaren sociale steun, en obstakels. Daarna spitst de inventarisatie zich toe per gezin, aansluitend bij eerdere informatie en actueel ontwikkelingsmoment. De informatie over de opvoedingssituatie wordt gearchiveerd in het JGZ-dossier. Het DMO-protocol wordt beschouwd als een veelbelovende werkwijze voor de vroegsignalering van een stagnerende ontwikkeling op grond van risico’s in de brede opvoedingssituatie; de effectiviteit is echter nog onvoldoende bekend (Hermanns et al., 2005)(ZonMw, 2006).  Tan et al. onderzochten de betrouwbaarheid van het DMO-protocol binnen een aantal consultatiebureaus (Tan, 2005). De betrouwbaarheid werd als ‘goed’ beoordeeld met voor de vijf risicodomeinen Cronbach’s alfa’s in de range 0,864-0,964. De externe validiteit werd bepaald op de kindleeftijd van 8 weken door vergelijking van de resultaten van het DMO-protocol met ervaren opvoedingsstress (NOSI) en behoefte/ontvangen aan professionele, maatschappelijke en sociale steun (FNS). Correlaties van het DMO-protocol met de gevalideerde instrumenten varieerden van 0,33 tot 0,47, afhankelijk van het domein (‘rol partner’, ‘welbevinden primaire opvoeder’, ‘welbevinden kind’). Als mogelijke verklaring voor de zwakke correlaties noemen de onderzoekers de instabiliteit van de door ouders waargenomen opvoedingssituatie in de eerste levensmaanden. Tussen afname van de vragenlijsten en het vaststellen van een ‘problematische’ situatie volgens het DMO-protocol verstreek gemiddeld 7 dagen (oplopend tot ruim 2 weken). Ruim de helft van de ouders (55%) met een discrepantie tussen het resultaat van het DMO-protocol en vragenlijstscore gaf aan dat de situatie gewijzigd was in de tussenliggende periode. Vanwege deze instabiliteit verdient het aanbeveling om bij validatie een kortere periode aan te houden tussen het DMO-protocol en de vragenlijsten, en zo mogelijk herhaalde metingen uit te voeren.  Vergelijkend dossieronderzoek voor en na implementatie van het DMO-protocol gaf aan dat respectievelijk 18% en 33% van de gezinnen beschouwd werd als ‘problematisch’ (Tenhaeff, 2004). In het onderzoek van Tan et al. verschilden de gezinnen waar het DMO-protocol werd gebruikt in risicofactoren van de controlegroep (bijv. etniciteit, inkomen, opleidingsniveau. sociale steun), zodat hier geen valide vergelijking mogelijk was van de prevalentie van gesignaleerde problematiek tussen de werkwijze met en zonder DMO-protocol (Tan, 2005). Vooralsnog bestaat onvoldoende inzicht in de validiteit van het DMO-protocol als signaleringsinstrument/methodiek voor een stagnerende ontwikkeling of risicosituaties daarop.   | **RELEVANTIE** | | --- | | Het eerste onderdeel in het wettelijk verplichte, uniforme deel van het Basistakenpakket Jeugdgezondheidszorg (JGZ) is signalering en monitoring. Hierbij gaat het om “het systematisch volgen van ontwikkelingen in de gezondheidstoestand van een jeugdige en/of groepen jeugdigen. Daarnaast worden factoren in kaart gebracht die de gezondheid bevorderen of bedreigen” (VWS, 2002).  De huidige werkwijze van de JGZ 0-4 jaar is relatief medisch-, kind- probleem- en momentgericht. Het DMO-protocol biedt ten opzichte van ieder van deze vier aspecten een bepaalde meerwaarde. Mede daarom werd het DMO-P door de Inventgroep als veelbelovende aanpak voor de JGZ aangemerkt (Hermanns et al., 2005). De meerwaarde van het DMO-protocol vergeleken met de huidige werkwijze binnen de JGZ 0-4 jaar wordt gevormd door de volgende kenmerken.  Ten eerste is het DMO-protocol ook systematisch psychosociaal gericht waarbij de gehele opvoedingssituatie wordt beschouwd. De huidige werkwijze in de JGZ 0-4 jaar beperkt zich veeleer tot signalering en monitoring van ontwikkelingskenmerken van het individuele kind zoals vastgesteld door Gesell (Brouwers-de Jong, 1996) met het van Wiechenonderzoek. Het DMO-protocol is gericht op het kind binnen zijn/haar context. Volgens het bio-ecologisch model (Bronfenbrenner, 2005) dat ten grondslag ligt aan het DMO-protocol, vindt ontwikkeling door continue wisselwerking tussen kind en omgeving. Naast de individuele ontwikkeling van het kind hecht het DMO-protocol groot belang aan de monitoring van de opvoedingsomgeving met het doel hier zonodig invloed op uit te oefenen ten behoeve van de ontwikkeling van het kind.  Ten tweede is in vergelijking met de huidige werkwijze het DMO-protocol minder gericht op manifeste problematiek bij het kind. De traditionele kindgerichtheid van de JGZ 0-4 jaar hangt samen met een zekere probleemgerichtheid van de praktijkvoering. Binnen de traditionele praktijkvoering komt interventie vrijwel altijd tot stand naar aanleiding van manifeste problematiek in de ontwikkeling van het kind Bij het DMO-protocol staat de brede opvoedingssituatie centraal als risicofactor, waardoor interventie in een eerder stadium (op risicosituaties) meer voor de hand ligt. Hiermee is het DMO-protocol meer gericht op preventie dan de traditionele werkwijze. Door de continue monitoring van de opvoedingssituatie is het DMO-protocol meer dan de huidige werkwijze gericht op risicoprocessen in plaats van een momentopname. Kind en context vormen tesamen een dynamisch systeem die elkaar in positieve en negatieve zin kunnen beïnvloeden. Het DMO-protocol sluit hiermee aan bij het advies van de Inventgroep om gezinnen systematisch te volgen (Hermanns et al. 2005). De traditionele JGZ 0/4 jaar werkt overwegend screenend, in de zin dat op één bepaald moment beoordeeld wordt of een kind wel of niet tot een bepaalde (risico)groep behoort.  Ten derde, is het DMO-protocol gericht op ondersteuning in plaats van advisering. Sinds duidelijk werd dat ouders ongevraagd advies minder positief ervaren (Caris, 1997) hebben veel thuiszorgorganisaties ingezet op vraaggericht werken. In vervolg hierop beoogt het DMO-protocol ´empowerment´ in de zin van proactief competentie verhogen door ouders positief te activeren tot formuleren van ‘vragen’ en zelf vinden van oplossingen. Ouders worden als gelijkwaardige partners beschouwd en hun perceptie van eventuele van de opvoedingssituatie staat centraal. Eerder onderzoek heeft aangetoond dat de mening van ouders voorspellend is voor toekomstig gedragsproblematiek (Rothbart & Bates, 1998). Bovendien zijn interventies effectiever als de mening van ouders sturend is (Turnbull, 2000).  Tot slot sluit het DMO-protocol aan bij de CARE-Nl, een instrument/methodiek ter inschatting van risicosituaties voor kindermishandeling en –verwaarlozing dat geïmplementeerd wordt binnen de Bureaus Jeugdzorg en Advies- en Meldpunten Kindermishandeling (AMK’s). Beide instrumenten gaan uit van het bio-ecologisch ontwikkelingsmodel (Meyers, 1999)(Booth, 2002)(Bronfenbrenner, 2005). Met het DMO-protocol wordt een doorgaande systematiek ontwikkeld zoals past binnen het programma Samen Starten. Het Centrum Jeugdgezondheid van het Rijksinstituut voor Volksgezondheid en Milieuhygiëne (RIVM) heeft Samen Starten en het DMO protocol aanbevolen voor gebruik in de JGZ 0-4 jaar.  In bredere context draagt het voorgestelde project bij aan de academisering van de JGZ. Het draagt bij aan de ontwikkeling van ‘evidence-based public health’ door wetenschappelijke onderbouwing van een voor de JGZ relevante interventie. Door de inbedding van het onderzoek binnen de reguliere JGZ worden professionals betrokken bij voor hen relevant wetenschappelijk onderzoek. |  DOELSTELLING Doel van het voorgestelde project is het bepalen van de effectiviteit van het DMO-protocol voor signalering van sociaal-emotionele problematiek en risico’s daarop vanuit opvoedingssituatie.  A] Vraagstellingen betreffende de validatie van het DMO-protocol:  1. Wat is de voorspellende waarde van door de JGZ gesignaleerde (dreigende) sociaal-emotionele problematiek in de eerste 15 levensmaanden?  2. Wat is de overeenkomst tussen signalering van problematische situaties op de domeinen van het DMO-protocol en beleving hiervan zoals gemeten met gestandaardiseerde meetinstrumenten?  3. Wat is de voorspellende waarde van de bevinding op elk domein van het DMO-protocol afzonderlijk en in combinaties voor sociaal-emotionele problematiek op de leeftijd van 18 maanden? |

B] Vraagstellingen betreffende vergelijking van de werkwijze met en zonder DMO-protocol:

1. Hoe groot is het verschil in proportie gesignaleerde sociaal-emotionele problematiek in de eerste 15 levensmaanden?

2. Hoe groot is het verschil in proportie gesignaleerde sociaal-emotionele problematiek op de leeftijd van 18 maanden?

3. Hoe groot is het verschil in de proportie juist en onjuist gesignaleerde sociaal-emotionele problematiek en risicosituaties daarop bij vergelijking met internationaal geaccepteerde standaardtesten?

# ONDERZOEKSOPZET

Binnen een quasi-experimentele studie wordt het DMO-protocol gevalideerd. Bij de fasering van implementatie van het DMO-protocol binnen de instelling voor JGZ 0-4 jaar Icare zijn twee vergelijkbare regio’s geselecteerd wat betreft sociaaldemografische kenmerken, waarbij in de ene regio het DMO-protocol is geïmplementeerd bij aanvang van het project (‘DMO-implementatieregio’) terwijl dit nog niet het geval is in de andere regio (‘DMO-controleregio’). Deze quasi-experimentele setting biedt de gelegenheid de signalering van (dreigende) sociaal-emotionele problematiek te vergelijken tussen beide regio’s. Gezinnen worden geïncludeerd gedurende de eerste acht levensweken en gedurende 18 maanden gevolgd op het ontstaan van sociaal-emotionele problematiek en risicosituaties daarop vanuit de opvoedingssituatie. De inclusieperiode bedraagt 12 maanden. Binnen dit quasi-experiment wordt een validatiestudie uitgevoerd waarbij gesignaleerde problematiek wordt vergeleken met referentietesten die de vijf domeinen van het DMO-protocol omvatten, i.e. functioneren/welbevinden van het kind, functioneren/welbevinden van de primaire verzorger/opvoeder, rol van de partner, ervaren sociale steun, en obstakels. Voor ieder gezin met gesignaleerde problematiek worden twee gezinnen uitgenodigd zonder gesignaleerde problematiek uit hetzelfde deel van het werkgebied (dus ‘DMO-implementatieregio’ of ‘DMO-controleregio’). De instroom voor deze geïntegreerde validatiestudie heeft plaats in de eerste 15 levensmaanden.

**SETTING.** Jeugdgezondheidszorg is preventieve gezondheidszorg die aan alle kinderen van nul tot negentien jaar in Nederland wordt aangeboden. Het doel van de jeugdgezondheidszorg is het volgen van de fysieke, sociale, psychische en cognitieve ontwikkeling van kinderen en het signaleren van stoornissen daarin, gericht op het tijdig aanbieden van interventies.

Het onderzoek wordt uitgevoerd tijdens de reguliere consulten van het consultatiebureau, i.e. JGZ 0-4 jaar. De opkomst naar deze bureaus is in deze leeftijdsgroep binnen de voorgestelde onderzoekregio’s boven de 95%. Binnen Icare wordt het DMO-protocol in het hele werkgebied geïmplementeerd. Bij aanvang van de implementatie is rekening gehouden met een effectiviteitsonderzoek zoals hier voorgesteld. Daartoe zijn twee vergelijkbare regio’s geselecteerd wat betreft sociaaldemografische kenmerken. In een regio is het DMO-protocol geïmplementeerd (‘DMO-implementatie regio’s’) en de andere regio’ staat op de wachtlijst voor implementatie (‘DMO-controleregio’). Dit betekent dat bij aanvang van het voorgestelde effectiviteitonderzoek de JGZ-medewerkers reeds getraind zijn in de ‘DMO-implementatieregio’ en ervaring hebben opgedaan met deze werkwijze. In de ‘DMO-controleregio’ is ‘care as usual’ gehandhaafd.

De implementeerbaarheid van het DMO-protocol als zodanig valt buiten het bestek van deze studie en maakt deel uit van een lopend onderzoeksvoorstel naar de implementatie van SamenStarten (tevens koppeling aan Stevig Ouderschap). Bij aanvang van dit project heeft implementatie reeds plaatsgehad. Dit impliceert een uitbreiding van de consulttijd (per kind) met tenminste 15 minuten voor het eerste verpleegkundige consult (± 8 weken postnataal) waarin de opvoedingssituatie uitgebreid met ouders besproken wordt. Deze 15 extra minuten zijn financieel reeds gedekt door de gemeenten waar met het DMO-protocol gewerkt wordt door JGZ- Icare. Ook het dossier is aangepast met een formulier voor notatie van bevindingen volgens het DMO-protocol. Voor dossiervorming middels het EKD heeft Icare reeds de nodige stappen ondernomen om ook hierin het DMO-dossierformulier op te nemen.

**IMPLEMENTATIE DMO-PROTOCOL.**

Voor de implementatie hebben medewerkers een training gevolgd, bestaande uit een groepsbijeenkomst van één dag en minimaal twee individuele begeleidingsgesprekken. Tijdens de groepsbijeenkomst worden medewerkers geschoold in de theoretische achtergronden en het systeem. De individuele begeleiding bestaat uit analyse van minimaal twee opgenomen gesprekken van de JGZ-professional, samen met een Video Interactie Coach (verder te noemen VIC). Inhoudelijke kennis, verbale en non-verbale gesprekstechnieken worden op gestandaardiseerde wijze geëvalueerd volgens criteria uit de Richtlijnen Gesprekstechnieken (Tan, 2006). Leervragen en fragmenten ter illustratie van deze leervragen worden door de JGZ-medewerker geformuleerd aan de hand van het Evaluatieformulier Video-Opname. Afhankelijk van de vorderingen wordt bepaald of meer begeleidingsgesprekken nodig zijn. Jaarlijks wordt de toepassing van het DMO-protocol geëvalueerd aan de hand van een analyse van een nieuwe opname van een gesprek.

**SIGNALERING IN DE ‘DMO-IMPLEMENTATIE REGIO.** De werkwijze volgens het DMO-protocol is sterk gestructureerd en vastgelegd in een gedetailleerde handleiding (Tan, 2006). Signalering met hulp van het DMO-protocol gebeurt door hiervoor getrainde JGZ-professionals (verpleegkundigen en artsen). Volgens het protocol vindt op de kindleeftijd van 8 weken een uitgebreide inventarisatie plaats van risico’s in de brede opvoedingssituatie, geoperationaliseerd als vijf risicodomeinen: functioneren/welbevinden van het kind, functioneren/welbevinden van de primaire verzorger/opvoeder, rol van de partner, ervaren sociale steun, en obstakels. Bij vervolggesprekken worden veranderingen in de opvoedingssituatie geïnventariseerd. Aan het eind van ieder gesprek komen ouder en JGZ-professional tot een gezamenlijke conclusie over de opvoedingsituatie, omschreven als: ‘probleem’, ‘zo-zo’, of ‘prima’, waarbij ‘probleem’ impliceert dat de ouder(s) behoefte hebben aan ondersteuning. Op grond daarvan wordt in samenspraak met de ouder(s) zo nodig tot interventies besloten.

**SIGNALERING IN DE ‘DMO-CONTROLEREGIO.** In de ‘DMO-controleregio’ is de wijze van consultvoering volgens ‘care as usual’. Hierbij gaat het om “het systematisch volgen van ontwikkelingen in de gezondheidstoestand van een jeugdige en/of groepen jeugdigen. Daarnaast worden factoren in kaart gebracht die de gezondheid bevorderen of bedreigen” (VWS, 2002). Na ieder consult wordt de JGZ-professional gevraagd schriftelijk aan te geven of er vermoeden is van sociaal-emotionele problematiek of een verhoogd risico daarop gezien de opvoedingssituatie (2-3 vragen). De vragenlijsten worden aansluitend naar het onderzoekcentrum gestuurd voor verwerking. De vragenlijst wordt niet opgenomen in het dossier zodat de JGZ-professional bij volgende consulten geblindeerd is voor informatie die is verstrekt t.b.v. het onderzoek.

**SELECTIE VOOR AANVULLEND ONDERZOEK.** Tijdens het postnatale huisbezoek (kindleeftijd 2-3 weken) krijgen ouders schriftelijke en mondelinge informatie over het onderzoek. Toestemming wordt gevraagd bij een van de volgende bezoeken aan het consultatiebureau zodat ouders bedenktijd hebben. Deze toestemming wordt verkregen vóór de uitgebreide inventarisatie van de opvoedingssituatie op de leeftijd van 8 weken. Vanaf de leeftijd van 8 weken wordt bij elk bezoek aan het consultatiebureau het risico beoordeeld op (dreigende) sociaal-emotionele problematiek. In de ´DMO-implementatieregio´ gebeurt deze signalering op basis van het DMO-protocol; in de ‘DMO-controleregio’ volgens de traditionele wijze van consultvoering. De populatie voor het aanvullend onderzoek betreft een dynamisch cohort waarbij gezinnen op ieder regulier JGZ-contactmoment tussen de leeftijd van 8 weken en 12 maanden kunnen instromen, namelijk bij 2, 3, 4, 6, 9, en 11 maanden. Bij signalering van een ‘problematisch’ gezin op enig regulier JGZ-contactmoment wordt het gezin door de JGZ-medewerker uitgenodigd voor aanvullend onderzoek. In geval van discrepantie tussen de beoordeling van de JGZ-medewerker (‘problematisch’) en de ouders (‘zo-zo’ of ‘prima’) zal de situatie worden behandeld als ‘problematisch’ en volgt uitnodiging voor aanvullend onderzoek. Voor ieder ‘problematisch’ gezin nodigt de JGZ-medewerker uit hetzelfde werkgebied twee ‘niet-problematische‘ gezinnen uit met een kind van dezelfde leeftijd en geslacht. In de stedelijke gebieden zijn de werkgebieden zijn wijkgericht, wat matching op sociaal-economische positie mogelijk maakt. Op het platteland zijn de werkgebieden georganiseerd naar woonkern wat dezelfde mogelijkheden geeft.

| |  | | --- | |  | |
| --- | --- | --- |

Figuur 1 geeft het protocol weer vanuit het perspectief van de ouders. Voor alle ouders is er één contactmoment op de leeftijd van 8 weken voor deelname aan het onderzoek, voor alle deelnemers één contactmoment op de leeftijd van 18 maanden (invullen van de CBCL), en voor een selectie van de deelnemers (242 ‘problematische’ en 486 ‘niet-problematische’ gezinnen) één maal aanvullend onderzoek.

**Figuur 1. Inclusieschema**

1 wk: uitnodiging

8 wk: toestemming, 72%

N = 5000

N = 3600

+

-

18 m: eindmeting CBCL

N = 3600

Uitgenodigd

Deelname, 85%

N = 3060

2–12m: problematische situatie

N = 360

N = 3240

+

-

+

-

Deelname, 70%

Aanvullend onderzoek

N = 242

N = 118

N = 486

N = 2754

Uitgenodigd

N=170

N=72

N=340

N=146

+

-

+

-

# STUDIE POPULATIE EN UITNODIGING TOT DEELNAME

Alle gezinnen met een pasgeboren kind die woonachtig zijn in de onderzoeksregio’s worden uitgenodigd om aan het onderzoek deel te nemen. De ouder(s) of verzorger(s) worden geïnformeerd over het onderzoek door de JGZ-professional tijdens het postnatale huisbezoek (kindleeftijd 2 weken). Naast de mondelinge toelichting ontvangen de ouder(s) of verzorger(s) een informatiebrief en een informatiefolder. In de informatiefolder wordt de opzet en de doelstellingen van het onderzoek toegelicht. Toestemming wordt gevraagd bij een van de volgende bezoeken aan het consultatiebureau zodat ouders bedenktijd hebben (kindleeftijd 4 weken of 8 weken). Deze toestemming wordt verkregen vóór de uitgebreide inventarisatie van de opvoedingssituatie op de leeftijd van 8 weken. Na de toestemming vullen zij het schriftelijke toestemmingsformulier in. Naast enkele kenmerken van de gezinnen (aantal kinderen, jongen/ meisje, eenouder gezin ja/nee) worden de redenen van ouders of verzorgers om af te zien van deelname door JGZ-professionals geregistreerd.

**Populatie.** Alle gezinnen met een pasgeboren kind die woonachtig zijn in de onderzoeksregio’s worden uitgenodigd om aan het onderzoek deel te nemen.

**In- en exclusie criteria.** Ouders met onvoldoende mondelinge beheersing van de Nederlandse taal worden uitgesloten van deelname.

## Steekproefgrootte

Eerder onderzoek bij 2-4 jarigen in Nederland laat zien dat de jeugdgezondheidszorg bij 10-12% van de kinderen sociaal-emotionele problematiek signaleert. Van de gesignaleerde kinderen heeft 22-23% ook een verhoogde (afwijkende) score op een referentietest (Child Behavior CheckList) (Reijneveld, 2004). De voorspellende waarde van door de JGZ gesignaleerde problematiek, zonder gebruikmaking van het DMO-protocol, stellen we daarmee op 22-23%. De voorspellende waarde van een JGZ-oordeel ‘afwezigheid van sociaal-emotionele problematiek’ is 92-93%. Voor de werkwijze met DMO-protocol wordt een verhoging van de voorspellende waarde van een positieve test met 20% relevant gesteld. Bij een power van 80% en een alfa van 0.05 zijn daarvoor in zowel de ‘DMO-implementatieregio’ als de ‘DMO-controleregio’ 85 ‘problematische’ signaleringen nodig, met ieder 170 ‘niet-problematische’ signaleringen. Uitgaande van 70% deelname worden voor aanvullend onderzoek uitgenodigd 121 ‘problematische’ en 243 ‘niet-problematische’ signaleringen in de ‘DMO-implementatieregio’ en de ‘DMO-controleregio. Hiermee kan de voorspellende waarde van een negatieve test met 5% precisie bepaald worden op 92%.

Op basis van geboortestatistieken worden ruim 2500 geboortes verwacht in zowel de ‘DMO-implementatieregio’ als de ‘DMO-controleregio’ (CBS-Statline). Bij een verwachte deelname van 72% aan het onderzoek betekent dit 1800 gezinnen in elke regio. Bij een cumulatieve incidentie van 10% in de periode tussen geboorte en leeftijd van 15 maanden betekent dit 180 ‘problematische’ signaleringen in zowel de ‘DMO-implementatieregio’ als de ‘DMO-controleregio’. Dit is voldoende om bij 121 gezinnen uit te nodigen voor aanvullend onderzoek in de verwachting dat van 85 gezinnen (70%) de dataverzameling compleet is (zie ook figuur 1: inclusieschema).

## Terugtrekking van deelname

Ouders kunnen op elk moment zonder opgave van redenen hun deelname aan de studie deels of volledig beëindigen. Dit wordt door de verpleegkundige of consultatiebureau arts aan de onderzoeker of onderzoeksmedewerker doorgegeven.

## Vroegtijdige beëindiging van de studie

Op basis van de powerberekening (zie 4.1) lijkt het niet aannemelijk dat de doelstellingen van de studie niet worden behaald door een te kleine instroom.

# METHODE

## Procedures

**Aanvullend onderzoek**

Het aanvullend onderzoek zal binnen één week na het JGZ-contactmoment worden uitgevoerd tijdens een huisbezoek voorafgaande aan een door de JGZ aangeboden interventie. Hiertoe zullen onderzoeksassistenten getraind worden die niet betrokken zijn bij de reguliere JGZ-zorg. Onafhankelijk van het JGZ-contactmoment waarop een ‘problematische’ gezinssituatie wordt geconstateerd, bestaat het aanvullend onderzoek uit internationaal geaccepteerde en gevalideerde vragenlijsten over de vijf domeinen van het DMO-protocol:

- Ontwikkeling: Ages & Stages Questionnaire voor Social-Emotional development (ASQ-SE).
- Sociale Steun: Eenzaamheidscore, Sociale Steun Lijst-Interacties (SSL).
- Rol partner / gezinsfunctioneren: Family Assessment Device (FAD), te ontwikkelen lijst naar tevredenheid over rol partner bij verzorging & opvoeding.
- Competentie primaire verzorger: Depression-Anxiety-Stress-Scales (DASS), Problem Setting & Behavior Checklist (PSCB), Nijmeegse Ouderlijke Stress Index-Kort (NOSIK).
- Obstakels:Vragenlijst van Rots-deVries (2002) over materiële basisvoorwaarden voor een gezonde ontwikkeling en opvoeding van het kind.

**Eindmeting**

Als een kind 18 maanden oud is, volgt de eindmeting bij alle gezinnen die toestemming gaven op de leeftijd van 8 weken in zowel de ‘DMO-implementatieregio’ als de ‘DMO-controleregio’, bij gezinnen die wel en niet zijn uitgenodigd voor aanvullend onderzoek. Hiertoe krijgen ouders voorafgaande aan het contactmoment de Child Behavior CheckList (CBCL) toegestuurd met het verzoek deze ingevuld mee te nemen, of deze samen met een getrainde onderzoeksassistent in te vullen tijdens het JGZ-contactmoment. Het invullen van de CBCL duurt ca. 20 minuten. Met deze procedure werd in eerder onderzoek een deelname van 92% gehaald (Zeijl et al., 2005). Afname van de CBCL op de leeftijd van 18 maanden vormt tevens een basis voor verder vervolgonderzoek.

**Responsebevordering**

Binnen het onderzoek worden maatregelen genomen om deelname van risicogroepen te bevorderen. Het aanvullend onderzoek vindt plaats tijdens een mondeling interview gedurende een persoonlijk huisbezoek. Er zullen hoge eisen gesteld worden aan de communicatieve en sociale vaardigheden van de onderzoeksassistent die de huisbezoeken zal uitvoeren. Voorafgaande aan het huisbezoek wordt door de onderzoeksassistent die het huisbezoek uitvoert telefonisch contact met de ouders opgenomen om de afspraak te verifiëren en het aanvullend onderzoek nogmaals kort toe te lichten. De afspraak voor het huisbezoek zal worden gemaakt door de JGZ-medewerker bij wie de ouders bekend zijn, aansluitend aan het bezoek aan het consultatiebureau. De JGZ-medewerkers spelen een belangrijke rol bij de rekrutering van de onderzoeksdeelnemers. Zij zullen als onderdeel van het project worden getraind in het motiveren van gezinnen voor het onderzoek (en hen vervolgens vast houden). Om de deelname aan de eindmeting op de leeftijd van 18 maanden zo hoog mogelijk te houden, zal de kinderen een verjaardagskaart worden toegezonden, en krijgen ouders die aan het aanvullend onderzoek deelnamen een klein presentje. Gezinnen die deelname weigeren aan het onderzoek of een van de onderdelen (aanvullend onderzoek, eindmeting) zal worden gevraagd naar de reden; tevens worden achtergrondkenmerken geïnventariseerd.

## Meetinstrumenten

Metingen betreffen de sociaal-emotionele ontwikkeling van het kind en risicovolle situatie hiervoor. De metingen worden uitgevoerd bij de primaire opvoeder. De keuze van de instrumenten is gebaseerd op validatie, acceptatie voor de cliënt, en gebruik in eerder onderzoek ter vergelijking. Vragen over de sociaal-emotionele ontwikkeling van het kind en risicosituaties daarvoor (opvoedcompetentie, sociale steun, rol partner, obstakels) kunnen emotioneel belastend zijn voor ouders en hierdoor bias veroorzaken in de verkregen informatie. Om dit te ondervangen is gekozen voor gebruik van meerdere instrumenten voor deze domeinen. Hierna volgt een beschrijving van de meetinstrumenten per domein van het DMO-protocol.

***Welbevinden kind / sociaal-emotionele ontwikkeling.***

Wat betreft het welbevinden van het kind focust het DMO-protocol zich op de sociaal-emotionele ontwikkeling. Hiertoe worden twee instrumenten gebruikt. 1] Ages & Stages Questionnaire for Socio-Emotional development (ASQ-SE) (Squires et al., 2002): een internationaal gevalideerd instrument van 30 items voor kinderen van 6-60 maanden oud die items bevat over gedrag op het gebied van zelfregulatie, volgzaamheid, communicatie, adaptief gedrag, autonomie, emoties en sociale enteracties. Voor kinderen jonger dan 6 maanden zijn op dit moment geen instrumenten beschikbaar voor het signaleren van sociaal-emotionele problematiek. 2] Child Behavior CheckList (CBCL) (Achenbach 2000) de internationaal gevalideerde standaard voor gedrags- en emotionele problematiek vanaf 18 maanden. De CBCL wordt afgenomen voor alle deelnemende kinderen, dus alle geborenen in de inclusieperiode van 12 maanden waarvan de ouders toestemming geven voor deelname aan het onderzoek (schatting N=3600).

***Sociale Steun.***

1] Eenzaamheidscore (11 items) (deJong-Gierveld & Kamphuis, 1985), een gevalideerde lijst die de intensiteit van gemis bepaalt en gebruikt wordt in de Monitor Volksgezondheid. 2] Sociale Steun Lijst-Interacties en negatieve items, SSL-I en SSL-N (41 items) (van Sonderen, 1994) , een lijst naar ervaren sociale steun. Rol van de partner 1] Family Assessment Device (FAD) (12 items) (Epstein et al., 1983), een internationaal gevalideerde lijst naar ervaren emotionele relaties en functioneren binnen het gezin. 2] Een te ontwikkelen vragenlijst naar tevredenheid van de primaire verzorger/opvoeder over de rol van de partner bij verzorging- en opvoedingstaken.

***Competentie van de primaire verzorger.***

1] Depression-Axiety-Stress-Scales (DASS) (42 items), een internationaal gevalideerde instrument voor depressie en angst (Lovibond & Lovibond, 1995). Het gaat hier om de meting van algemene gevoelens van angst en depressie zonder specificatie naar aanleiding of setting. De vragenlijst wordt veel gebruikt in onderzoek naar relaties met opvoedingsgedrag van ouders en interventies daarop. 2] Problem Setting & Behavior Checklist (PSCB) (28 items), een internationaal gevalideerde vragenlijst die het vertrouwen meet van de primaire verzorger in het omgaan met problemen zoals driftbuien etc in gangbare situaties met het kind zoals aankleden, meenemen naar buiten, boodschappen doen, etc. (Sanders & Woolley, 2005) 3] Parental Stress Index (PSI), een internationaal gevalideerde vragenlijst van 30 items die de stress van de primaire verzorger meet bij het opvoeden/verzorgen van het kind (in Nederland bekend als NOSI) (Abidin, 1997).

***Obstakels.***

1) Een lijst van stellingen die de relatie weergeeft tussen financiële middelen en basisvoorwaarden voor de gezondheid van het kind, zoals voeding, ontwikkelingsstimuli (speelgoed, boeken, kinderwagen) (Rots-de Vries, 2002) Algemeen Naast vragenlijsten over de domeinen van het DMO-protocol wordt de ouders gevraagd naar algemene achtergrondkenmerken als opleiding, werk, inkomen, etniciteit, woonomstandigheden, gezinsstructuur. Verder wordt informatie verzameld over behandeling van de kinderen ivm ontwikkelingsproblematiek, bijvoorbeeld vanwege vroeggeboorte, verstandelijke beperking, sensorische of motorische beperking.

# STATISTISCHE ANALYSE

Vraagstellingen m.b.t. de validatie van het DMO-protocol worden geanalyseerd door:

a. Bepaling van de sensitiviteit, specificiteit, positief en negatief voorspellende waarden van signalering van (dreigende) sociaal emotionele problematiek in vergelijking tot de ASQ-SE (6-15mnd).

b. Berekening van Kappawaarden als maat van overeenkomst tussen problematiek op een bepaald domein van het DMO-protocol en de relevante referentietesten van door de ouders ervaren problematische situaties (c.q. risicosituaties).

Vraagstellingen m.b.t. de vergelijking tussen de werkwijze met DMO-protocol (‘DMO-implementatieregio’) en de traditionele werkwijze (‘DMO-controleregio’) worden geanalyseerd door:

a. Vergelijking van de proporties gesignaleerde sociaal-emotionele problematiek en risicosituaties daarvoor door chi2-testen en logistische regressie (bij controle voor verschillen in populatiekenmerken uit de ‘DMO-implementatieregio’ en de ‘DMO-controleregio’).

b. Vergelijking van de sensitiviteit, specificiteit, positief en negatief voorspellende waarden van signalering van (dreigende) sociaal emotionele problematiek in vergelijking tot de ASQ-SE.

c. Vergelijking van de proporties gedrags- en emotionele problematiek op de leeftijd van 18 maanden middels chi2-testen en logistische regressie. Gepoolde analyse van deelnemers in de ‘DMO-impelentatieregio’ en de ‘DMO-controleregio’: a] Logistische regressie met berekening van de odds ratio en 95% betrouwbaarheidsinterval als associatiemaat tussen problematiek op de domeinen van het DMO-protocol en sociaal-emotionele problematiek gemeten met de ASQ-SE (6-15mnd), en de CBCL (18mnd).

# ETHISCHE ASPECTEN

## Regulation statement

Deze studie zal worden uitgevoerd volgens de principes die zijn vastgelegd in de Declaration of Helsinki (Tokyo 2004) en in overeenstemming met de Wet Medisch-wetenschappelijk Onderzoek met mensen (WMO).

## Werving van deelnemers en informed consent

De ouder(s) of verzorger(s) worden geïnformeerd over het onderzoek door de JGZ-professional tijdens het postnatale huisbezoek (kindleeftijd 2-3 weken). Naast de mondelinge toelichting ontvangen de ouder(s) of verzorger(s) een informatiebrief en folder. In de informatiefolder wordt de opzet en de doelstellingen van het onderzoek toegelicht. Toestemming wordt gevraagd bij een van de volgende bezoeken aan het consultatiebureau zodat ouders bedenktijd hebben. Deze toestemming wordt verkregen vóór de uitgebreide inventarisatie van de opvoedingssituatie op de leeftijd van 8 weken. Na de toestemming vullen zij het schriftelijke toestemmingsformulier in.

## Verzekering

In de aanbiedingsbrief hebben we de METC verzocht om een ontheffing van de verzekeringsplicht aangezien wij geen schade door dood of letsel van de deelnemers verwachten gedurende en na de deelname van de personen aan het onderzoek.

## Incentives

Ouders en de JGZ-professionals ontvangen een klein presentje voor hun inspanningen voor het onderzoek. Daarnaast zullen we ouders en JGZ-professionals informeren over de onderzoeksresultaten. Om de deelname aan de eindmeting op de leeftijd van 18 maanden zo hoog mogelijk te houden, zal de kinderen een verjaardagskaart worden toegezonden, en krijgen ouders die aan het aanvullend onderzoek deelnamen een klein presentje.

# ADMINISTRATIEVE PROCEDURES

## Documentatie en toegankelijkheid van de data

Onderzoeker(s) en onderzoeksmedewerkers mogen geen informatie met elkaar uitwisselen over de gezinnen die zij ten behoeve van het aanvullend onderzoek spreken.

Alle onderzoeksgegevens worden vertrouwelijk behandeld en opgeslagen met een onderzoekscode zonder persoonlijke details zoals naam of adres. Het bestand met de persoonlijke gegevens van de deelnemers, inclusief de onderzoekscode is apart gearchiveerd en beveiligd. Alleen de betrokken onderzoeker, onderzoeksmedewerkers en projectleider hebben toegang tot dit databestand. Het bestand met de persoonlijke gegevens wordt na afloop van het onderzoek vernietigd.

De verantwoordelijkheid voor de opslag van de data en de beveiliging is de verantwoordelijkheid van de projectleider, wie eventueel derden inschakelt om de beveiliging te garanderen (o.a. ICT-medewerkers).

Alle onderzoeksgegevens worden uitgebreid gecontroleerd en fouten worden gecorrigeerd. Missende gegevens worden nagevraagd bij ouders of JGZ-professionals.

## Publicaties

In wetenschappelijke zin draagt het project bij aan de ontwikkeling van standaarden voor de opsporing van risico’s voor sociale-emotionele ontwikkeling van jonge kinderen. Resultaten van het project zijn daarmee relevant voor financiers, beleidsmakers en uitvoerders van onderzoek en zorg (preventief en curatief) op de gebieden gezondheid, gedrag en opvoeding. Voor onderzoekers vindt kennisoverdracht plaats middels presentaties tijdens wetenschappelijke bijeenkomsten, publicaties in nationale en internationale wetenschappelijke tijdschriften, en een wetenschappelijke dissertatie. Voor individuele zorgverleners vindt kennisoverdracht plaats middels publicatie in relevante tijdschriften en andere bronnen, en presentaties op locatie en tijdens nascholingsdagen.

# REFERENTIES

Abidin DP. Parenting Stress Index: a measure of the prent-child system. In: Zalaquett C, Wood R eds. Evaluating stress: a book of resources. Lanham, MD: Scarecrow Press, 1997:277-91.

Achenbach, T.M. en L.A. Rescorla (2000). Manual for the aseba preschool forms & profiles.Burlington, VT: University of Vermont, Research Center for Children, Youth, & families.

Bleeker J, Cornips MC, de Meer G, Reijneveld SA. Het Noordelijk Consortium Publieke Gezondheid: lokale preventie en vangnet zorg verbeteren (in Dutch). TSG 2006;84:358-9.

Bronfenbrenner U, Making human beings human: Bioecological perspectives on human development. 2005.

Booth A, Carver K, Granger DA. Biosocial perspectives on the family. J Marriage Fam 2000;62:1018-34.

Durlak A, Wells AM. Evaluation of indicated preventive intervention (secondary prevention) mental health programs for children and adolescents. Am J Community Psychol 1998;26:775-802.

Egger HL, Angold A. Common emotional and behavioral disorders in preschool children: presentation, nosology, and epidemiology. J Child Pscychol Psychiatr 2006;47:313-37.

Epstein, N.B., Baldwin, L.M., Bishop, D.S. (1983) The McMaster Family Assessment Device. Journal of Marital and Family Therapy, 9(2), 171-180.

Eijck S. Sturingsadvies deel 1 en 2. Koersen op het kind. Kompas voor het nieuwe kabinet. Den Haag: Ministerie van VWS, 2006. Gilsing R, Keuzenkamp R. Naar een stelsel van sociale indicatoren voor het Integraal Toezicht Jeugdzaken. Den Haag: SCP, 2004.

Hermanns J, Öry F, Schrijvers G (Inventgroep). Helpen bij opgroeien en opvoeden: eerder, sneller en beter. Een advies over vroegtijdige signalering en interventies bij opvoed- en opgroeiproblemen. Utrecht: Juliuscentrum, 2005.

Jong-Gierveld J de, Kamphuis FH. The development of a Rasch-type loneliness-scale. Applied Psychological Measurement, 1985; 9: 289-299.

Landry SH. Does early responsive parenting have a special importance for children’s development? Develop Psychol 2001;37:387-403. Lovibond SH, Lovibond PF. Manual for the Depression Anxiety Stress Scales. Sydney, NSW Australia: Psychology Foundation of Australia.

Meyers SA. Mothering in context: Ecological determinants of parent behaviour. Merill Palmer Quart 1999;45:332-57.

Nelson G, Westhues A, Macleod J. A meta-analysis of longitudinal research on preschool prevention programs for children. Prevention and Treatment 2003;6:31.

Reijneveld SA, Brugman E, Verhulst FC, Verloove-Vanhorick SP. Identification and management of psychosocial problems among toddlers in Dutch Preventive Child Health Care. Arch Pediatr Adolesc Med 2004;158:811-7.

Rots-de Vries MC, Koresbergen HT. Bedreiging van de gezondheid van kinderen door armoede: beleid en interventie. TSG 2002;80:275-81.

Rutter M. The interplay of nature, nurture, and developmental influences: The challenge ahead for mental health. Arch Gen Psych 2002;59:996-1000.

Sanders, MR, & Woolley ML. The relationship between global, domain and task-specific self-efficacy and parenting practices: Implications for parent training. Child: Care, Health and Development 2005; 31: 65–73.

Squires J, Potter LW, Bricker D :The ASQ User's Guide for the Ages and Stages Questionnaire: A parent - completed, child monitoring system. 2nd edition. Maryland, Baltimore, Paul Brookes Publishing Comp; 1990.

Tan N, van den Boom DC, Hermanns JJM. Protocol ter ondersteuning van de sociaal-emotionele ontwikkeling. Een volgsysteem voor consultatiebureaus (0-4 jarigen) ontwikkeld in opdracht van DMO. Eindverslag. Amsterdam: Universiteit van Amsterdam, 2005.

Tan N. Protocol DMO. Draaiboek Training Samen Starten. Amsterdam, 2006. VWS. Basistakenpakket Jeugdgezondheidszorg. Den Haag: Ministerie van VWS, 2002.

Van Sonderen, E.,. Het meten van sociale steun met de Sociale Steun Lijst-Interacties (SSL-I) en Sociale Steun Lijst-Discrepanties (SSL-D): Een handleiding. In: NCG reeks meetinstrumenten: 2 (Ed. Sanderman, R.,

Van Sonderen, E.). 1994, Groningen, NCG.

[www.samenstarten.nl](http://www.samenstarten.nl/)

[www.zonmw.nl](http://www.zonmw.nl/)

Zeijl E, Crone M, Wiefferink K, Keuzenkamp S, Reijneveld M. Kinderen in Nederland. Den Haag / Leiden: SCP / TNO, 2005.
